# Structural alteration of neurons in schizophrenia and its relation with auditory hallucination

**DOI:** 10.1192/j.eurpsy.2026.10163

**Published:** 2026-02-06

**Authors:** Ryuta Mizutani, Rino Saiga, Yoshiro Yamamoto, Chie Inomoto, Hiroshi Kajiwara, Yu Kakimoto, Masahiro Yasutake, Masayuki Uesugi, Akihisa Takeuchi, Kentaro Uesugi, Yasuko Terada, Yoshio Suzuki, Viktor Nikitin, Francesco De Carlo, Youta Torii, Itaru Kushima, Norio Ozaki, Shuji Iritani, Makoto Arai, Ken-ichi Oshima, Masanari Itokawa

**Affiliations:** 1 Department of Bioengineering, Tokai University, Japan; 2 RIKEN SPring-8 Center, Japan; 3 Department of Mathematics, Tokai University, Japan; 4 Department of Pathology, Tokai University School of Medicine, Japan; 5 Department of Forensic Medicine, Tokai University School of Medicine, Japan; 6 Japan Synchrotron Radiation Research Institute (JASRI/SPring-8), Japan; 7 Photon Factory, High Energy Accelerator Research Organization (KEK), Japan; 8 Advanced Photon Source, Argonne National Laboratory, USA; 9 Department of Psychiatry, Nagoya University Graduate School of Medicine, Japan; 10 Okehazama Hospital Fujita Mental Care Center, Japan; 11 Tokyo Metropolitan Institute of Medical Science, Japan; 12 Tokyo Metropolitan Matsuzawa Hospital, Japan

**Keywords:** hallucination, nano-CT, neuron, schizophrenia, structure

## Abstract

**Background:**

Volumetric changes in the superior temporal gyrus and anterior cingulate cortex have been repeatedly reported in studies on schizophrenia. Tractography and functional magnetic resonance imaging studies have suggested that alterations in connectivity involving the superior temporal gyrus and the anterior cingulate cortex are relevant to psychotic symptoms of schizophrenia.

**Methods:**

We analyzed nanometer-scale three-dimensional structures of brain tissues of the superior temporal gyrus and the anterior cingulate cortex in eight schizophrenia and eight control cases and evaluated structural parameters of their neurons. We then examined the relation between the neuronal parameters and clinical information including auditory hallucination score.

**Results:**

The obtained results indicated that 1) neurites become thin and tortuous in schizophrenia and that 2) somata become small in schizophrenia. The frequency distribution of neurite curvatures had a broad profile in the schizophrenia cases, whereas the control cases showed sharp peaks. In the scatter diagram of the standard deviation of neurite curvatures, the schizophrenia and control cases formed separate clusters, indicating that all 16 cases analyzed in this study can be assigned to either the schizophrenia or control group simply by using the diagram. The cingulate/temporal ratio of the standard deviation of neurite curvatures showed a strong positive correlation with the auditory hallucination score.

**Conclusions:**

The structural alteration of neurites observed in the schizophrenia cases should influence the function of affected brain areas by hindering communication between distant neurons. We suggest that the interplay of the temporal and cingulate cortices in the whole-brain network is relevant to auditory hallucination.

## Introduction

Structural changes in the brain have been repeatedly reported in studies on schizophrenia. Volumetric reduction in brain tissue as well as thinning of the cerebral cortex has been identified in multiple brain regions [1–11]. Volumetric changes in the superior temporal gyrus, which is involved in auditory function, have often been reported in schizophrenia [1, 4, 6, 7, 9, 10]. The anterior cingulate cortex, which is associated with emotional and cognitive functions [12, 13], is another area that has consistently shown volume changes in schizophrenia [3, 4, 9, 10].

Macroscopic changes in the brain have been studied in relation to schizophrenia symptoms. It has been reported that schizophrenia patients with auditory hallucinations had larger frontal and temporal cortical volumes than patients without auditory hallucinations [14], though another study reported that patients with auditory hallucinations show a significant volumetric decrease in the superior temporal gyrus [15]. Functional studies have suggested the involvement of multiple brain regions in auditory hallucinations. Altered activation in the superior temporal gyrus and anterior cingulate was shown to occur in schizophrenia patients with auditory hallucinations [16]. Recent tractography and functional magnetic resonance imaging studies have suggested that alterations in connectivity involving the superior temporal gyrus [17] and the anterior cingulate cortex [18] are relevant to psychotic symptoms of schizophrenia including auditory hallucinations. Therefore, we consider that the interplay of brain areas plays a crucial role in the development of schizophrenia symptoms.

The volumetric changes in gray matter are ascribable to cellular changes in the cerebral cortex. It has been suggested that the reduced cortical volume in schizophrenia can be explained by the reduced neuronal size rather than neuronal loss [19]. Indeed, a quantitative meta-analysis focused on neuron density indicated an increase in neuronal density [20]. Such a neuronal density increase without neuronal loss should be due to alterations in cellular structure, such as a reduction in interneuronal neuropil [21]. In order to delineate cellular changes in schizophrenia, it is imperative to investigate the three-dimensional microscopic structure of brain tissues of schizophrenia patients.

In this study, we analyzed nanometer-scale three-dimensional structures of post-mortem brain tissues of the Brodmann area 22 (BA22) of the superior temporal gyrus in four schizophrenia and four control cases. Structural parameters evaluated from the obtained neuronal structures along with our previous results on four other schizophrenia and four other control cases [22] revealed structural alterations of neurons in the BA22 area in schizophrenia. We previously reported an analysis on the BA24 area of the same 16 cases [23, 24]. The results for BA22 in this study and our previous findings for BA24 were examined to clarify the relationship between these regions. We also investigate the relation between the neuronal parameters and clinical information, including auditory hallucination score. This paper is thus a compilation of our nanometer-scale structural studies on the BA22 and BA24 tissues of the schizophrenia and control cases, covering about 14 years.

## Methods

### Cerebral tissues

All post-mortem human cerebral tissues were collected with informed consent from the legal next of kin using protocols approved by the Clinical Study Reviewing Board of Tokai University School of Medicine (application no. 07R-018) and the Ethics Committee of Tokyo Metropolitan Institute of Medical Science (approval nos. 17–18, 20–19 and 23–10), as reported previously [22–24]. This study was conducted under the approval of the Ethics Committee for the Human Subject Study of Tokai University (approval nos. 25009 and 25010). All experiments were performed in accordance with relevant guidelines and regulations. The number of cases subjected to the following analysis was determined from the experiment time available at synchrotron radiation facilities (Supplementary Table 1). Schizophrenia cases S1–S8 and control cases N1–N8 (Supplementary Table 2) are the same as those in our previous studies [22–24]. The schizophrenia cases were diagnosed according to the DSM-IV criteria by at least two experienced psychiatrists. Auditory hallucination scores of the schizophrenia cases were estimated as described previously [24] by using the Present State Examination (PSE) method [25].

BA22 tissues of the superior temporal gyrus were dissected from the left hemispheres of the autopsied brains of the schizophrenia and control cases. The dissected tissues were subjected to Golgi impregnation and embedded in epoxy resin according to the method reported previously [23].

### Synchrotron radiation experiments

The overall structure of the resin-embedded brain tissue samples was visualized with simple projection microtomography at the BL20XU beamline [26] of SPring-8. The experimental conditions are summarized in Supplementary Table 1. Absorption contrast images were collected using a visible-light conversion type X-ray imaging detector consisting of a phosphor screen, optical lenses, and complementary metal-oxide semiconductor (CMOS) imaging detectors (ORCA-Flash2.8 and ORCA-Flash4.0, Hamamatsu Photonics, Japan), as reported previously [23]. Tomographic slices were reconstructed from the X-ray images of the brain tissues and stacked to obtain their three-dimensional images, which were then used to examine the laminal cytoarchitecture.

Next, individual neurons in layer V were visualized with synchrotron radiation nanotomography (nano-CT) equipped with Fresnel zone plate optics [27]. The nano-CT experiments were performed at the BL37XU [28] and the BL47XU [29] beamlines of the SPring-8 synchrotron radiation facility, and at the 32-ID beamline [30, 31] of the Advanced Photon Source (APS) of Argonne National Laboratory, as reported previously [22–24]. Supplementary Table 1 summarizes the experimental conditions. Spatial resolutions of the obtained images were evaluated from the Fourier domain plot [32] or by using three-dimensional test patterns [33].

#### Structural analysis

Tomographic slices were reconstructed from the obtained datasets with the convolution-back-projection method using the RecView software (RRID: SCR_016531) [34], as reported previously [23]. The tomographic reconstruction was performed by RS. The obtained three-dimensional image datasets were renamed randomly and provided to RM without case information to remove human biases in the structural analysis. RM built Cartesian coordinate models of the tissue structures by tracing the neuronal network in the three-dimensional image. The model building was conducted using the MCTrace software (RRID: SCR_016532) [35]. After finishing the model, RM reported the number of neurites in each structural model to RS. RS aggregated the number of neurites according to the case information. RS chose additional datasets to be analyzed so as to equalize the analysis amount between cases. The chosen datasets were returned to RM without the case information. These model building and aggregation steps were repeated in three batches. The first batch consisting of datasets S5A, S5B, S8A, S8C, S8E, N5A, N5C, and N5E (Supplementary Table 3) were analyzed together with mouse datasets reported in our previous study on mouse neurons [36]. Case information on these datasets were disclosed to RM after finishing the model of the first batch in order to report the mouse study [36]. The second batch consisted of the major part of the analyzed datasets: a total of 20 datasets of S5–S8 and N5–N8 cases. The third batch consisted of the remaining datasets, S6B, N6A, N6E, N8A, and N8C. After all the datasets of these second and third batches had been analyzed, the nano-CT experiment date of each dataset was disclosed to RM in order to correct the voxel width. Finally, the case information was disclosed in order to assign datasets to each case.

All 33 models built with the above procedure were subjected to structural analyses in accordance with the method reported previously [23, 36] by using the MCTrace software [35]. The analysis results are summarized in Supplementary Tables 2 and 3. The obtained structural parameters, along with our previous results on the BA24 area of the same cases [24] were evaluated in order to examine the structural characteristics of neurons.

#### Statistical tests

Statistical tests were performed using the R software. Significance was defined as *p* < 0.05. Mean soma length and standard deviation of neurite curvatures were analyzed using two-way ANOVA with group (schizophrenia/control) and brain area (BA22/BA24) as main factors. Results of multiple tests were corrected with the Bonferroni method. Correlations between structural parameters and clinical information were examined by using Pearson’s correlation coefficient.

## Results

### Structural characteristics of neurons in schizophrenia

**Figure 1.**
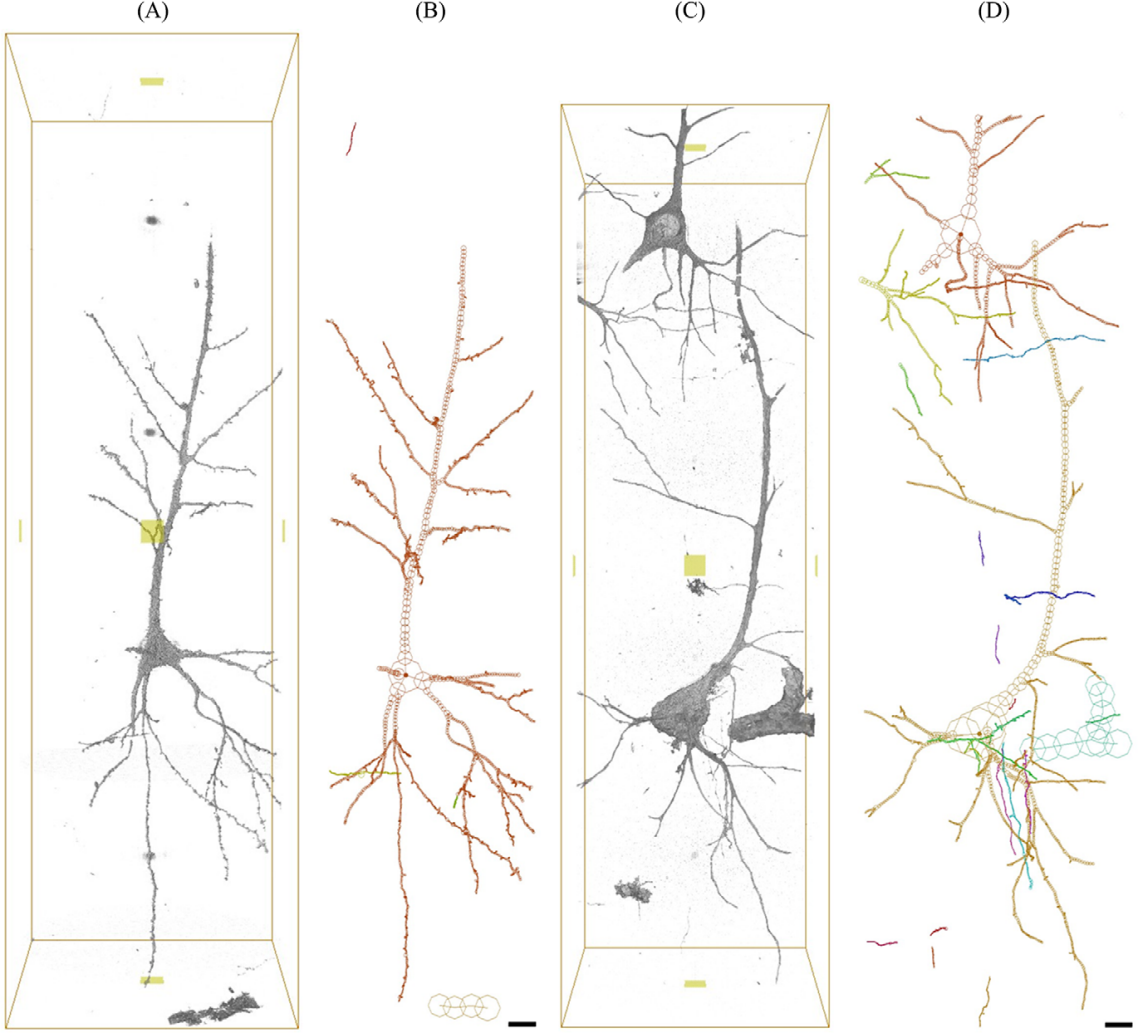
Three-dimensional structure of schizophrenia S8A and control N5A datasets. The pial surface is toward the top. Three-dimensional images were rendered with the Scatter HQ algorithm by using the VG Studio software (Volume Graphics). Voxel values from 80 to 800 were rendered. Cartesian coordinate models were drawn with the MCTrace software [35]. Structural constituents are color-coded. Nodes constituting the structure are indicated with octagons, and soma nodes with dots. Scale bars: 10 μm. (A) Rendering of a three-dimensional image of the S8A dataset. (B) Cartesian coordinate model of the S8A structure. (C) Three-dimensional image of the N5A dataset. (D) Cartesian coordinate model of the N5A structure.

Neurons in layer V of BA22 tissues of the superior temporal gyrus of four schizophrenia cases (S5–S8) and four control cases (N5–N8) were visualized with synchrotron radiation nanotomography (Supplementary Table 1), as reported previously [22–24]. The visualized nanometer-scale images were then reconstructed by tracing the neuronal networks in Cartesian coordinate space in order to analyze their three-dimensional structures (Figure 1; Supplementary Figures 1–3), as previously reported for four other schizophrenia (S1–S4) and four other control cases (N1–N4) [19]. The obtained BA22 structures consisted of 104 neurons, 5,083 neurites, and 22,222 dendritic spines for the eight schizophrenia cases, and 118 neurons, 4,190 neurites, and 15,683 dendritic spines for the eight control cases (Supplementary Tables 2 and 3).

**Figure 2.**
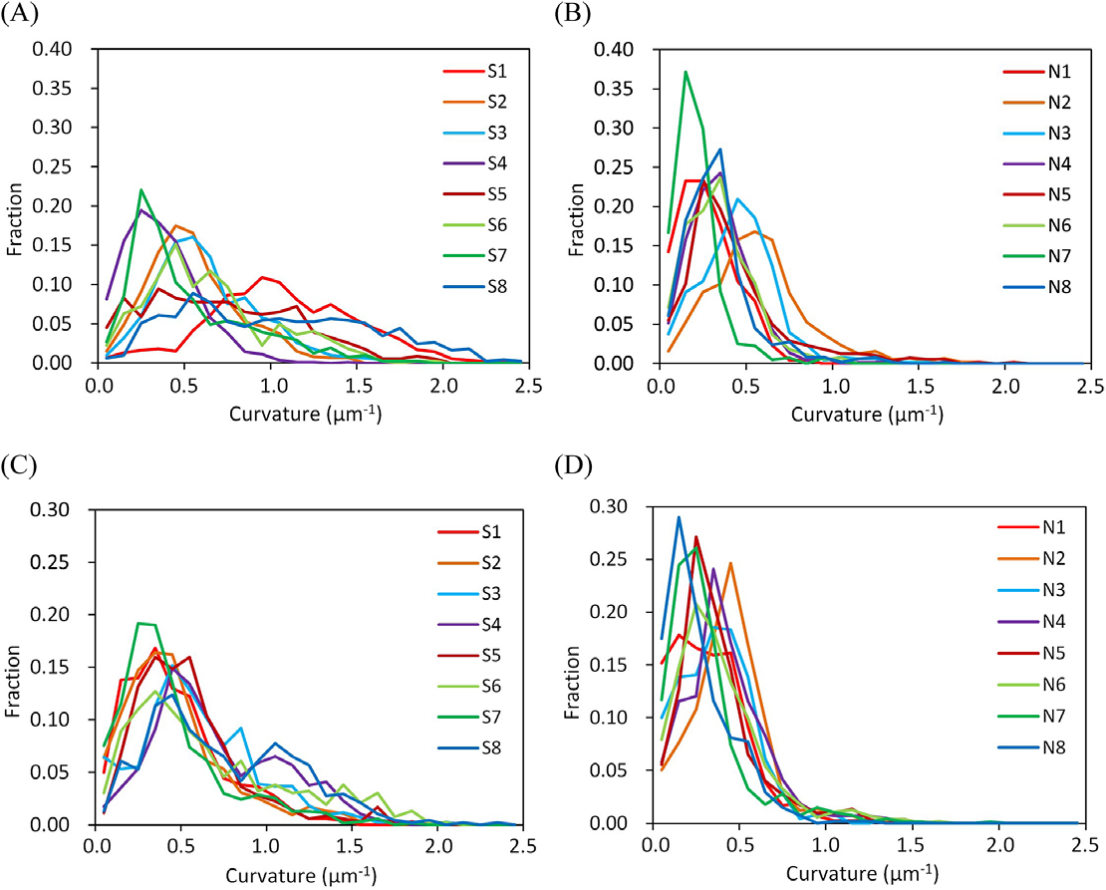
Frequency distributions of neurite curvatures. Frequency fraction in each 0.1 μm^−1^ bin of neurite curvature is plotted. Cases are color-coded and indicated with labels. (A) BA22 of schizophrenia cases. (B) BA22 of control cases. (C) BA24 of schizophrenia cases. (D) BA24 of control cases. Panels C and D are reproduced from ref [24].

Examples of the obtained structures and reconstructed models are shown in Figure 1. The three-dimensional images indicated that the neuronal somata of the schizophrenia cases are typically smaller than those of the controls. Another difference was observed in the spatial trajectory of neurites. The neurites of the schizophrenia cases are thin and tortuous, while those of the controls are rather straight and thick. In order to quantitatively delineate the structural differences between the schizophrenia and control cases, we evaluated structural parameters from the Cartesian coordinate models (Figure 1B,D) and examined their differences between the schizophrenia and control groups. The spatial trajectories of neurites can be regarded as three-dimensional curves that can be represented with two geometrical parameters called curvature and torsion. The curvature corresponds to the reciprocal of the radius of the trajectory curve and is inversely proportional to the neurite thickness diameter [23]. Therefore, a high neurite curvature represents a thin and tortuous neurite structure, whereas a low value indicates a thick and straight structure. We calculated the mean curvature for every neurite of the schizophrenia and control cases and plotted their frequency distribution (Figure 2). The obtained plot indicated that the schizophrenia cases have broad curvature distributions with long tails beyond 1.0 um^−1^ (Figure 2A), whereas the control cases showed sharp peaks with distributions showing little spread beyond 1.0 um^−1^ (Figure 2B). Similar profiles have been observed in BA24 tissues of schizophrenia cases (Figure 2C,D). The S8 case that had the distribution with the longest tail was reported to carry a chromosome 22q11.2 deletion [37]. These results indicate that the schizophrenia cases are characterized by a frequency distribution of neurite curvatures with a broad profile.

**Figure 3.**
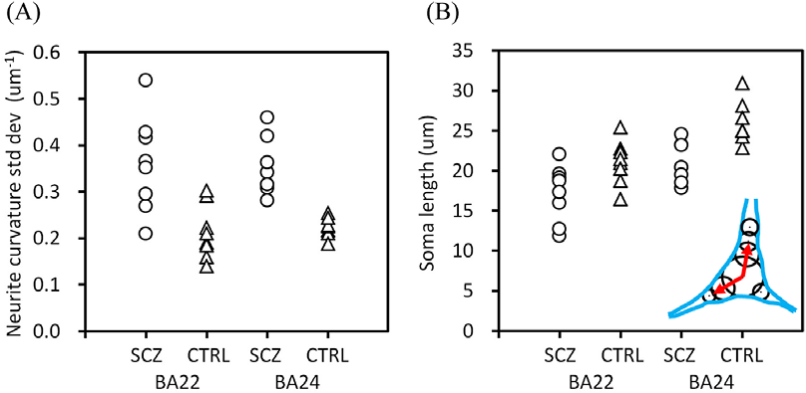
Differences in structural parameters. Schizophrenia cases are indicated with circles and controls with triangles. (A) Standard deviation of neurite curvatures showed a significant difference between the schizophrenia and control groups (*p* = 4.9 × 10^−6^, two-way ANOVA with group (schizophrenia/control) and brain area (BA22/BA24) as main factors, Bonferroni corrected). (B) Mean soma length showed differences between schizophrenia and control groups (*p* = 1.5 × 10^−4^) and between brain areas (*p* = 1.9 × 10^−4^). The definition of soma length [36] is indicated with red arrows in the inset.

The profile width of the frequency distribution of the neurite curvatures is represented with the standard deviation of the neurite curvatures. Figure 3A illustrates their plots. The standard deviation showed a significant difference between the schizophrenia and control groups (*p* = 4.9 × 10^−6^, two-way ANOVA with group (schizophrenia/control) and brain area (BA22/BA24) as main factors, Bonferroni corrected), while no significant difference was observed between the BA22 and BA24 areas. This confirmed that the schizophrenia cases have significantly more tortuous neurites than the control cases and hence, a broader distribution of neurite curvatures (Figure 2).

Another structural difference was found in regard to neuronal soma size. We defined the soma length as the sum of the lengths along the longitudinal soma shaft having diameters larger than half the maximum width [36], as shown in the inset of Figure 3B. The obtained mean soma length exhibited a significant difference between the schizophrenia and control groups (Figure 3B; *p* = 1.5 × 10^−4^, two-way ANOVA), and also between the BA22 and BA24 areas (*p* = 1.9 × 10^−4^). These results indicated that the schizophrenia cases have smaller neuronal somata than the control cases. The longer soma length in BA24 than in BA22 is consistent with the abundance of spindle-shape von Economo neurons in the anterior cingulate cortex [38].

### Relation between structural parameters of the BA22 and BA24 areas

**Figure 4.**
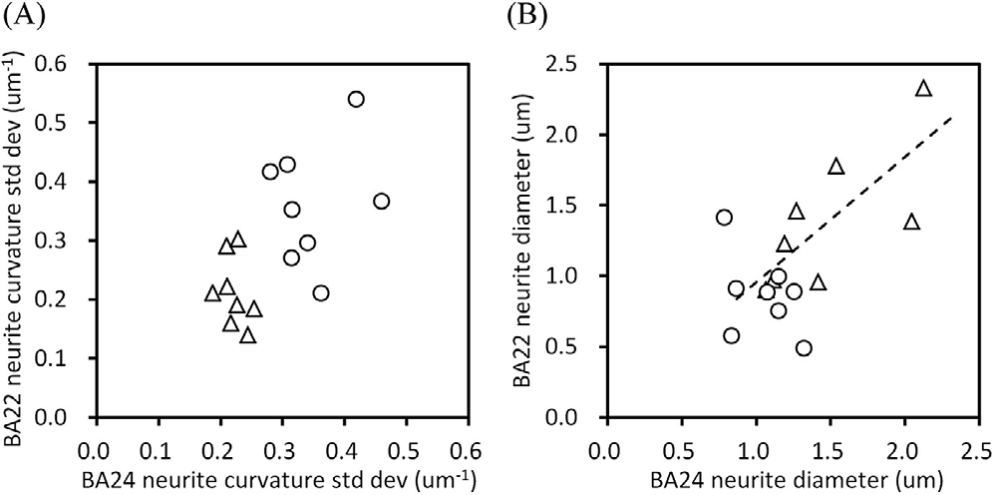
Structural relation between the BA22 and BA24 areas. Schizophrenia cases are indicated with circles and controls with triangles. (A) Scatter diagram of the standard deviation of neurite curvature. (B) Scatter diagram of neurite diameter. The dashed line is a linear regression for controls (Pearson’s *r* = 0.74, *p* = 0.035, *n* = 8).

Next, we examined the relation between the structural parameters of the BA22 and BA24 areas. Figure 4A shows a scatter diagram of the standard deviations of neurite curvatures of the BA22 and BA24 areas. The plots of the schizophrenia and control cases form separate clusters, indicating that all 16 cases analyzed in this study can be assigned to either the schizophrenia or control group simply by using this diagram. Figure 4B shows a scatter diagram of the mean neurite diameter of the BA22 and BA24 areas. The control cases exhibited a strong correlation between BA22 and BA24 (Pearson’s *r* = 0.74, *p* = 0.035, *n* = 8), indicating that these cerebral areas have nearly identical neurite diameters in the control cases. In contrast, the schizophrenia cases showed no obvious correlation between the areas. Some schizophrenia cases rather deviated from the correlation: the ratio of neurite diameter in BA24 to that in BA22 (hereafter called the BA24/BA22 ratio) of the schizophrenia group ranges from 0.56 (S4 case) to 2.7 (S1), while it is confined between 0.87 (N7) and 1.5 (N3) in the controls.

### Relation between neuronal structure and clinical parameters

**Figure 5.**
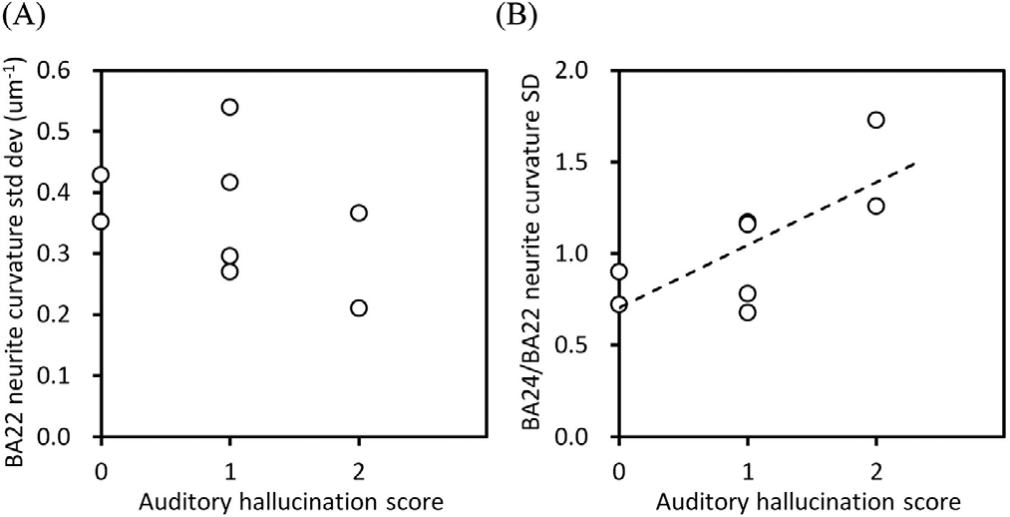
Relation between the structural parameters of schizophrenia cases versus their auditory hallucination score. (A) Standard deviation of neurite curvatures of BA22. (B) BA24/BA22 ratio of standard deviations of neurite curvatures. The dashed line is a linear regression (Pearson’s *r* = 0.73, *p* = 0.039, *n* = 8).

Clinical information of the schizophrenia and control cases are summarized in Supplementary Table 2. We examined the relation between the above-obtained structural parameters and the clinical parameters. The standard deviation of the neurite curvatures of BA22 showed a downtrend against the auditory hallucination score (Figure 5A), though no significant correlation was found between them. Moreover, other structural parameters of BA22, including the soma length, showed no significant correlation with the hallucination score (Supplementary Figure 4), indicating that the neuronal structure in the BA22 area itself has no clear link to the hallucination score. In contrast, the BA24/BA22 ratio of the standard deviation of neurite curvatures indicated a strong correlation to the auditory hallucination score (Figure 5B; Pearson’s *r* = 0.73, *p* = 0.039, *n* = 8). The BA24/BA22 ratio becomes large when the standard deviation of the neurite curvatures is high in BA24 and low in BA22. Therefore, the upper right end of this plot corresponds to the situation in which the neuronal structure in BA24 of the schizophrenia group is altered from that of the control group, but BA22s of both groups are structurally the same.

We also examined the relation between the structural parameters and the medication dose (Supplementary Table 2). The chlorpromazine-equivalent dose was plotted against the structural parameters of BA22 or BA24 (Supplementary Figure 5). No obvious relation was found between the medication dose and the structural parameters, indicating that medications administrated for these cases have no significant association with neuronal structure. Although the standard deviation of the neurite curvatures of BA24 exhibited a correlation with age for control cases [24], no obvious relation to age was observed for the structural parameters of BA22 (Supplementary Figure 6).

## Discussion

The nanometer-scale three-dimensional analysis of the cerebral tissues of schizophrenia and control cases indicated that neurites become thin and tortuous in schizophrenia, resulting in the frequency distribution of neurite curvatures having a broad profile (Figure 2A,C). According to cable theory [39], the thinning of neurites suppresses the transmission of action potentials between distal neurons. Hence, the structural alteration of neurites observed in the schizophrenia cases should influence the function of affected brain areas by hindering communication between distant neurons.

In the control cases, the mean neurite diameter of BA22 showed a strong correlation with that of BA24 (Figure 4B), suggesting that the neurite diameter is nearly constant regardless of the brain area. Since the structure of the temporal cortex is moderately heritable [40], its neuronal structures should also be heritable to some extent. In contrast, the neurite diameters of the schizophrenia cases did not show correlation between the cerebral areas (Figure 4B), indicating that neurite thickness is different between brain areas in some schizophrenia cases. This inter-area difference would impair the functional balance between the areas. It has been reported that schizophrenia patients tend to have a diverse profile of brain functional connectivity [41]. The lack of correlation between the neurite diameters of the BA22 and BA24 areas should thus affect the cooperation of these brain regions. We suggest that the BA22 and BA24 areas of the schizophrenia cases form a different relationship compared with the controls.

The scatter diagram of the standard deviations of the neurite curvatures of BA22 and BA24 (Figure 4A) separates all of the cases analyzed in this study into two groups on the basis of their neurite structures. This indicates that 1), in the schizophrenia cases, neurites undergo structural alteration in at least one of these areas, and that 2) all 16 cases can be distinguished as to whether they are schizophrenia or not by using the scatter diagram. We previously reported that the schizophrenia and control cases were plotted into separate groups in the scatter diagram of the standard deviation of neurite curvature versus neuronal soma length in BA24 [42]. These results mean that the neuronal structure is altered significantly in either the BA22 or BA24 area in the schizophrenia cases and the alteration thus aligns with their symptom-based DSM diagnosis of schizophrenia. Although the neuropathology of schizophrenia has long remained unrevealed [11, 19, 20, 43, 44], the results of these 16 cases suggest that schizophrenia is accompanied by distinct neuronal structural characteristics. Such characteristics should be further examined in terms of how they progress from pre-onset to post-onset periods.

The ratio of the standard deviations of the neurite curvatures showed a strong positive correlation with the auditory hallucination score (Figure 5B). This means that auditory hallucinations are likely to occur if BA24 is impaired and BA22 is normal, and are less likely to occur under the opposite condition. Our analysis also indicated that the BA22 parameters themselves showed no obvious relation with the hallucination score (Figure 5A). Indeed, studies on schizophrenia patients with auditory hallucinations reported both an increase [14] and decrease [15] in volume of the superior temporal gyrus. Therefore, auditory hallucination is not ascribable solely to the superior temporal gyrus. Alteration in brain-wide connectivity has rather been proposed as a underlying cause of schizophrenia symptoms including auditory hallucinations [45, 46]. The connectivity alteration involving the superior temporal gyrus and the anterior cingulate cortex should constitute part of the brain-wide dysconnectivity and, hence, be associated with auditory hallucination [17, 18]. We suggest that the interplay of the temporal and cingulate cortices in the whole-brain network is relevant to auditory hallucination.

The structural parameters of the BA22 and BA24 areas showed no obvious relation with medication dose (Supplementary Figure 5), indicating that the medications had little effect on neuronal alteration in schizophrenia. Therefore, the neuronal alterations observed in the schizophrenia cases should be targeted for novel treatments. We suggest that medications that can thicken neurites or enlarge neuronal somata are possible candidates for therapeutics for schizophrenia. One candidate would be neurotrophic factors that support neuronal growth, such as brain-derived neurotrophic factor (BDNF) and nerve growth factor (NGF). It has been reported that blood levels of the BDNF are reduced in schizophrenia [47] and that the BDNF expression influences cognitive impairments in schizophrenia [48]. The NGF serum concentration is also reported to be associated with gray matter volume in schizophrenia [49]. Another candidate to restore the neuronal shape would be cytoskeletal agents. The neuronal cytoskeleton has been proposed as a potential target for schizophrenia treatment [50]. The disrupted-in-schizophrenia 1 (DISC1) protein localizes to cytoskeleton-rich regions [51] and plays a role in the microtubule/centrosome cascade [52]. A recent study indicated that tubulin heterodimers are targeted by clozapine [53]. It may be possible to develop novel therapeutics by exploring these candidates.

Etiology of schizophrenia has been studied for several decades on the basis of brain structural changes [54–57]. Early brain imaging studies consistently reported ventricular dilations [54, 56] and volumetric reductions in the frontal and temporal cortices [55, 56]. Subsequent meta-analyses have confirmed reduced gray matter volume in multiple cortical areas, including the anterior cingulate cortex [3,4] and the superior temporal gyrus [4, 6, 7]. An umbrella review of 50 neuroimaging meta-analyses [57] also reported robust structural alterations, including the medial prefrontal cortex and the superior temporal gyrus. The neurite thinning and soma downsizing revealed in this study would be possible cellular mechanisms underlying the gray matter reduction in schizophrenia. Recent multimodal MRI studies reported progressive gray matter abnormalities during disorder course [58, 59]. Abnormalities in multiple brain areas, including auditory and language networks, have been identified as being associated with auditory hallucination [60]. In this study, a significant correlation was observed between the hallucination score and the BA24/BA22 ratio of the neurite-curvature standard-deviation (Figure 5B). This structo-functional correlation provides a clue to link the gray matter abnormalities to the auditory hallucination.

The primary limitation of this study is its analysis scale. Although this paper is a compilation of our studies made over the course of 14 years, its analysis is limited to the BA22 and BA24 areas of eight schizophrenia and eight control cases whose ethnicity were all Japanese. Further studies covering larger and different ethnic case/control groups should be conducted to examine the reproducibility of the results obtained in this study. The structural analysis of the BA22 and BA24 areas suggested that the interplay between these areas is relevant to schizophrenia symptoms (Figures 4 and 5B). However, the correlation between the neuronal structure and the auditory hallucination score requires replication, since the present analysis is limited to eight schizophrenia cases. The same structural analysis should be performed on other cerebral areas or brain regions to investigate the involvement of multiple brain domains in schizophrenia and to clarify responsible brain areas. Such studies will reveal the neuropathology of schizophrenia and consequently lead to the development of novel treatments for schizophrenia.

## Supporting information

10.1192/j.eurpsy.2026.10163.sm001 Mizutani et al. supplementary material 1 Mizutani et al. supplementary material

10.1192/j.eurpsy.2026.10163.sm002 Mizutani et al. supplementary material 2 Mizutani et al. supplementary material

## Data Availability

Structural parameters and case information are available in the supplementary materials. All other data will be provided after approval by the ethics committee.

## Supplementary material

The supplementary material for this article can be found at http://doi.org/10.1192/j.eurpsy.2026.10163.

## Analytic code availability

RecView software (RRID: SCR_016531) and its source code used for the tomographic calculation are available from https://mizutanilab.github.io under the BSD 2-Clause License. The MCTrace software (RRID: SCR_016532) used for model building and geometric analysis is available from the same site under the BSD 2-Clause License. Its source code is available upon request.
