# Supplementary material for: Structural alteration of neurons in schizophrenia and its relation with auditory hallucination
Source: Eur Psychiatry. 2026 Feb 6;69(1):e25. doi: 10.1192/j.eurpsy.2026.10163 (PMC12936874; doi:10.1192/j.eurpsy.2026.10163)
Supplement: Mizutani et al. supplementary material 1 — Mizutani et al. supplementary material [file S0924933826101631sup001.docx]

Supplementary Materials of:

**Structural alteration of neurons in schizophrenia and its relation with auditory hallucination**

Ryuta Mizutani^1,2^*, Rino Saiga^1^, Yoshiro Yamamoto^3^, Chie Inomoto^4^, Hiroshi Kajiwara^4^, Yu Kakimoto^5^, Masahiro Yasutake^6^, Masayuki Uesugi^6^, Akihisa Takeuchi^6^, Kentaro Uesugi^6^, Yasuko Terada^6^, Yoshio Suzuki^7^, Viktor Nikitin^8^, Francesco De Carlo^8^, Youta Torii^9^, Itaru Kushima^9^, Norio Ozaki^9^, Shuji Iritani^10^, Makoto Arai^11^, Ken-ichi Oshima^11,12^, Masanari Itokawa^11^

^1^Department of Bioengineering, Tokai University, Hiratsuka, Kanagawa 259-1292, Japan; ^2^RIKEN SPring-8 Center, Kouto, Sayo, Hyogo 679-5148, Japan; ^3^Department of Mathematics, Tokai University, Hiratsuka, Kanagawa 259-1292, Japan; ^4^Department of Pathology, Tokai University School of Medicine, Isehara, Kanagawa 259-1193, Japan; ^5^Department of Forensic Medicine, Tokai University School of Medicine, Kanagawa 259-1193, Japan; ^6^Japan Synchrotron Radiation Research Institute (JASRI/SPring-8), Sayo, Hyogo 679-5198, Japan; ^7^Photon Factory, High Energy Accelerator Research Organization (KEK), Tsukuba, Ibaraki 305-0801 Japan; ^8^Advanced Photon Source, Argonne National Laboratory, Lemont, IL 60439, USA; ^9^Department of Psychiatry, Nagoya University Graduate School of Medicine, Nagoya, Aichi 466-8550, Japan; ^10^Okehazama Hospital Fujita Mental Care Center, Toyoake, Aichi 470-1168, Japan; ^11^Tokyo Metropolitan Institute of Medical Science, Setagaya, Tokyo 156-8506, Japan; ^12^Tokyo Metropolitan Matsuzawa Hospital, Setagaya, Tokyo 156-0057, Japan

* ryuta@tokai.ac.jp; mizutanilaboratory@gmail.com

**Index**

Supplementary Figure 1 ... p. 2–7

Supplementary Figure 2 ... p. 8–13

Supplementary Figure 3 ... p. 14–20

Supplementary Figure 4 ... p. 21

Supplementary Figure 5 ... p. 22

Supplementary Figure 6 ... p. 23

Supplementary Tables 1–3 are provided separately.

**A B**


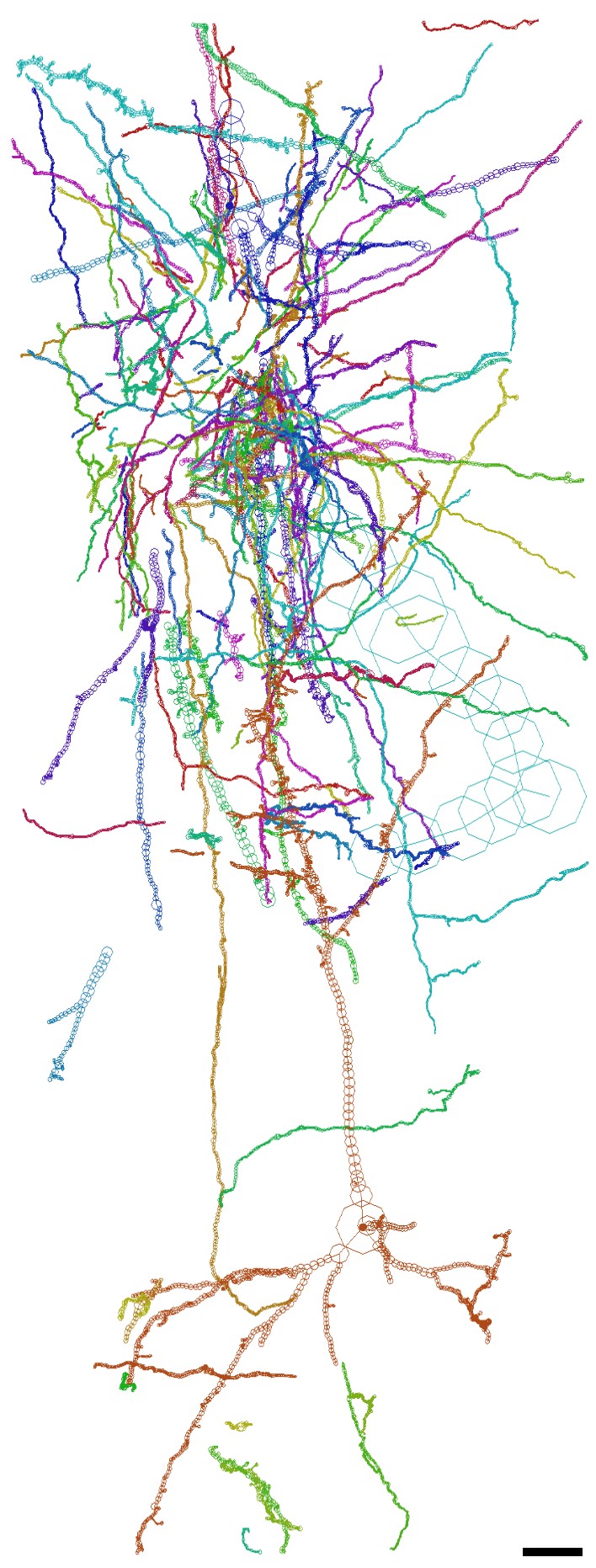

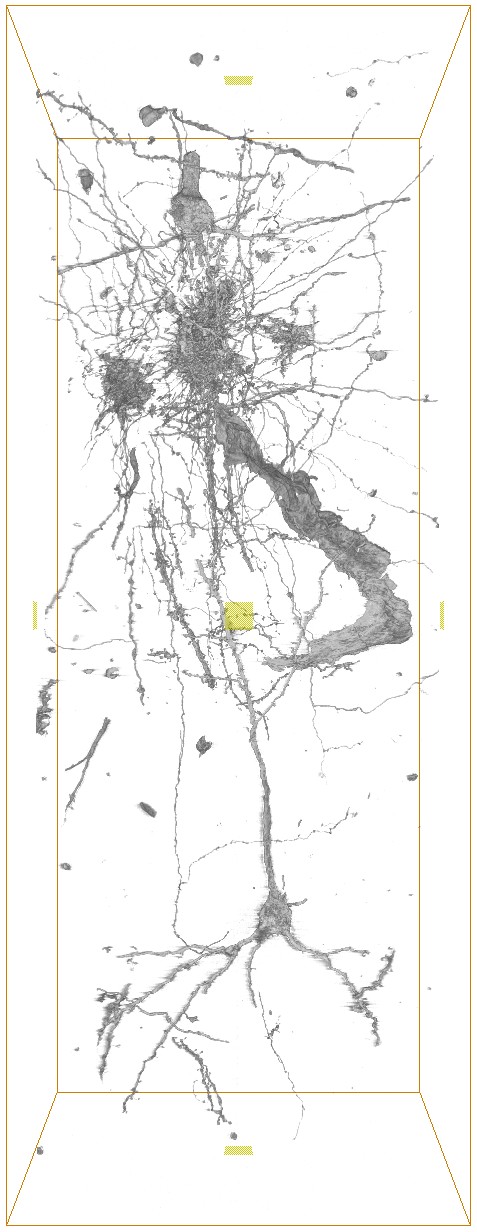


**Supplementary Figure 1.** Three-dimensional renderings of tissue structures and their Cartesian coordinate models. The pial surface is toward the top. The renderings were produced with the scatter HQ algorithm of the VG Studio software. The structural models were drawn with the MCTrace software. Model constituents are color-coded. Nodes constituting the model are indicated with octagons and soma nodes with dots. Scale bars: 10 μm. (**A**) Rendering of the S5A dataset of the schizophrenia S5 case. Voxel values from 80 to 800 are rendered. (**B**) Cartesian coordinate model of S5A.

**C D**


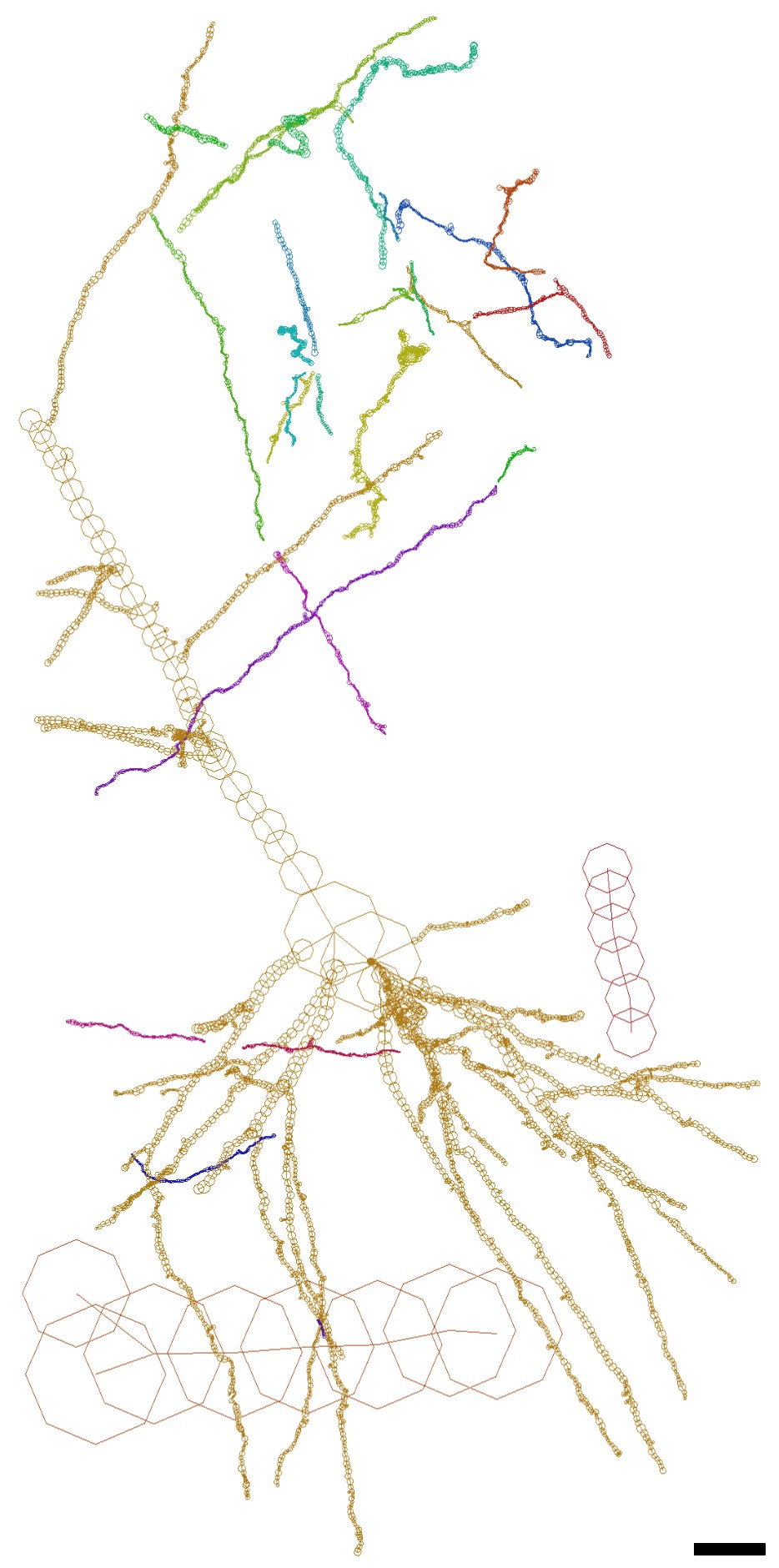

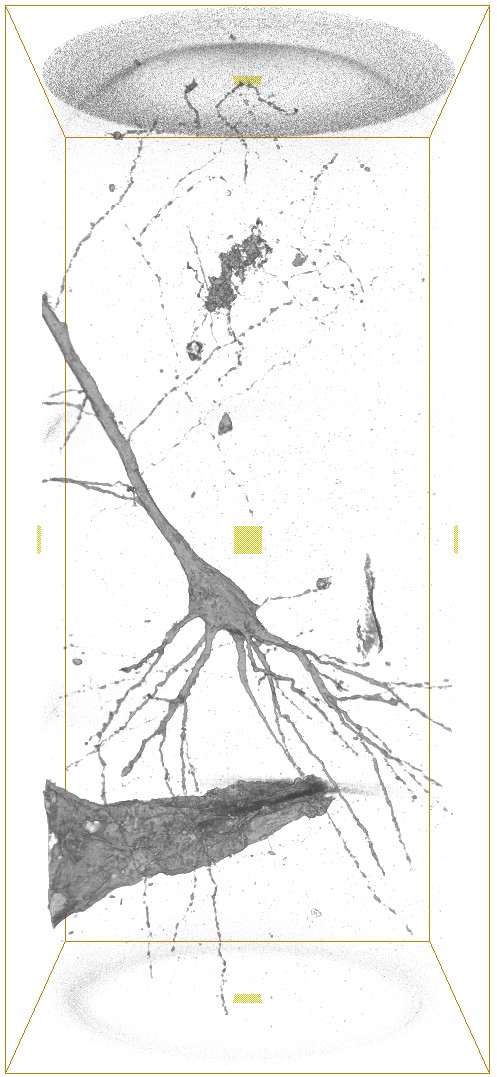


**Supplementary Figure 1 (cont'd).** Three-dimensional renderings of tissue structures and their Cartesian coordinate models. Scale bars: 10 μm. (**C**) Rendering of the S6A dataset of the schizophrenia S6 case. Voxel values from 80 to 800 are rendered. (**D**) Model of S6A.

**E F**


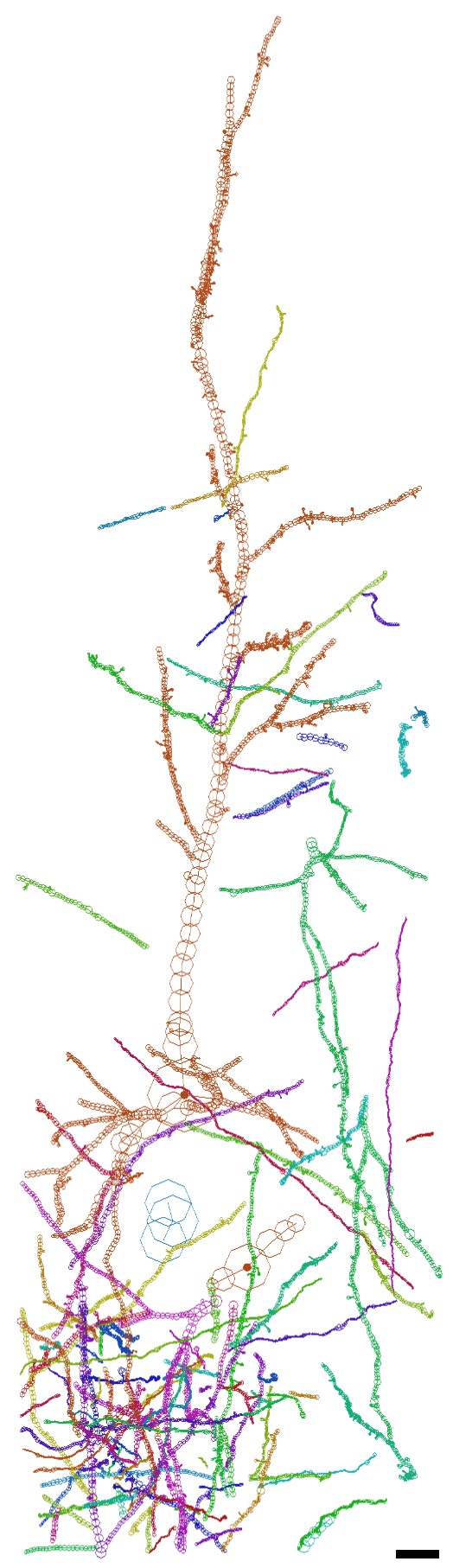

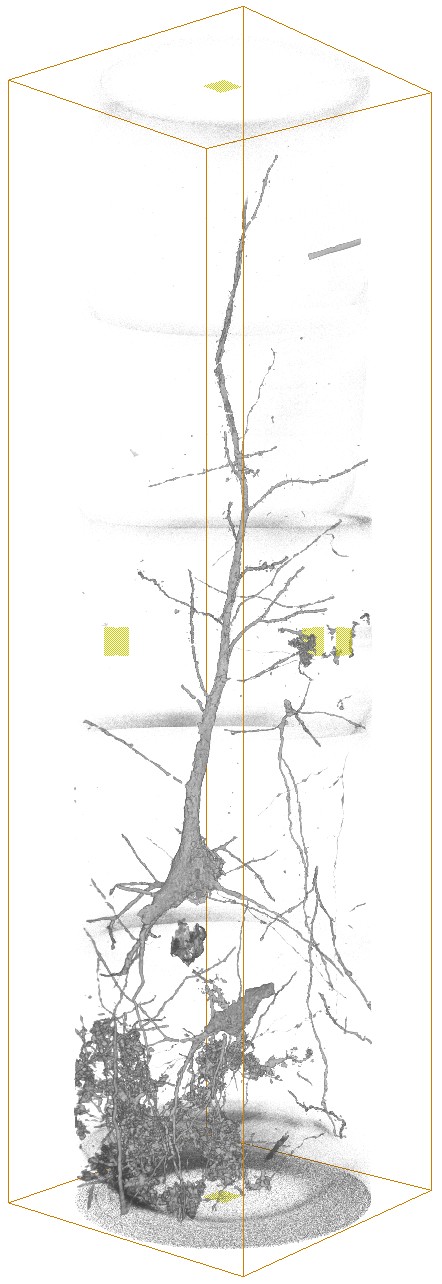


**Supplementary Figure 1 (cont'd).** Three-dimensional renderings of tissue structures and their Cartesian coordinate models. Scale bars: 10 μm. (**E**) Rendering of the S7A dataset of the schizophrenia S7 case. Voxel values from 80 to 800 are rendered. (**F**) Model of S7A.

**G H**


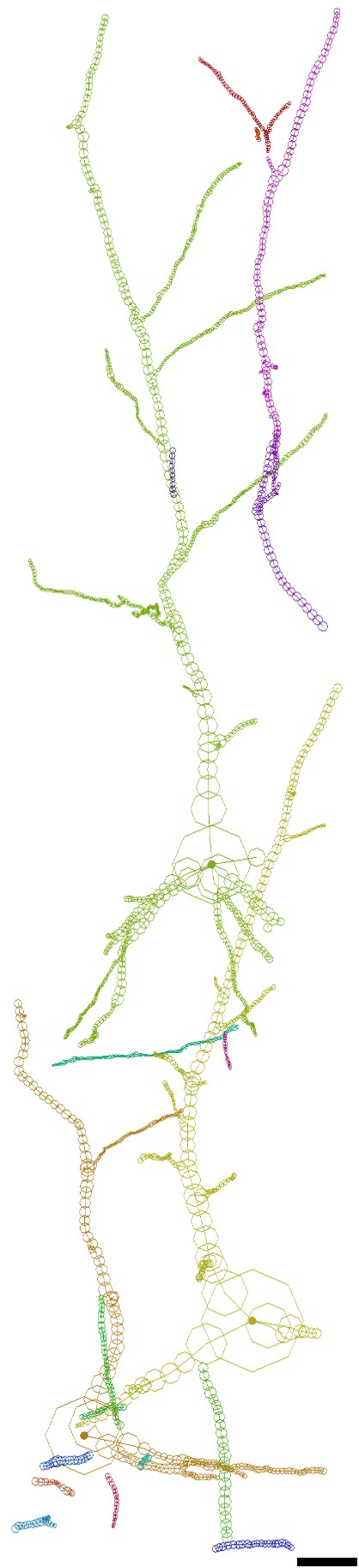

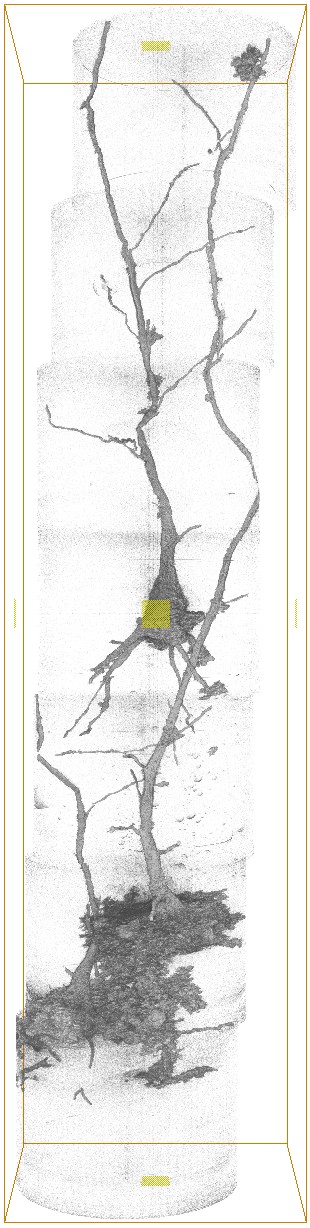


**Supplementary Figure 1 (cont'd).** Three-dimensional renderings of tissue structures and their Cartesian coordinate models. Scale bars: 10 μm. (G) Rendering of the N6A dataset of the schizophrenia N6 case. Voxel values from 50 to 500 are rendered. (**H**) Model of N6A.

**I J**


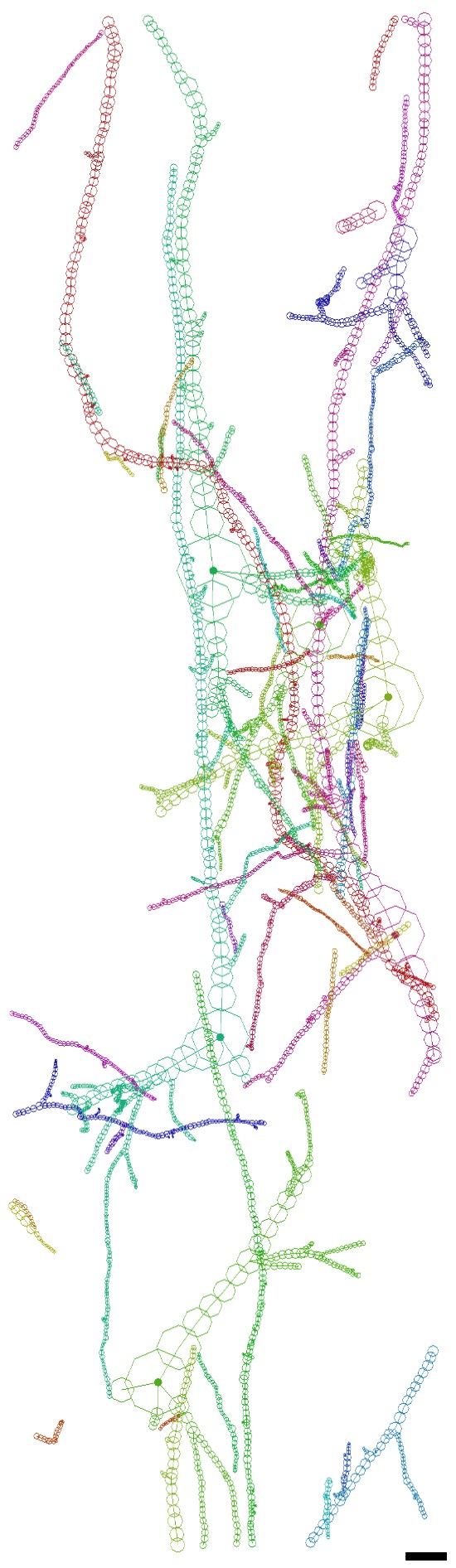

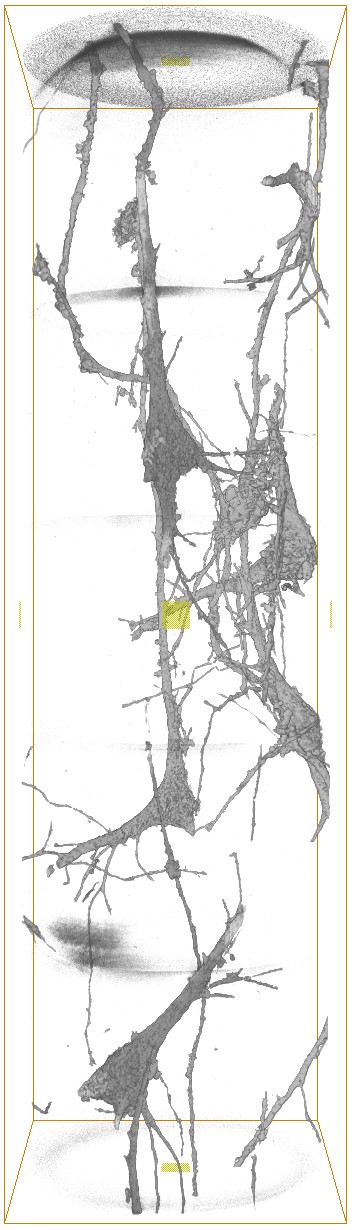


**Supplementary Figure 1 (cont'd).** Three-dimensional renderings of tissue structures and their Cartesian coordinate models. Scale bars: 10 μm. (**I**) Rendering of the N7A dataset of the schizophrenia N7 case. Voxel values from 80 to 800 are rendered. (**J**) Model of N7A.

**K L**


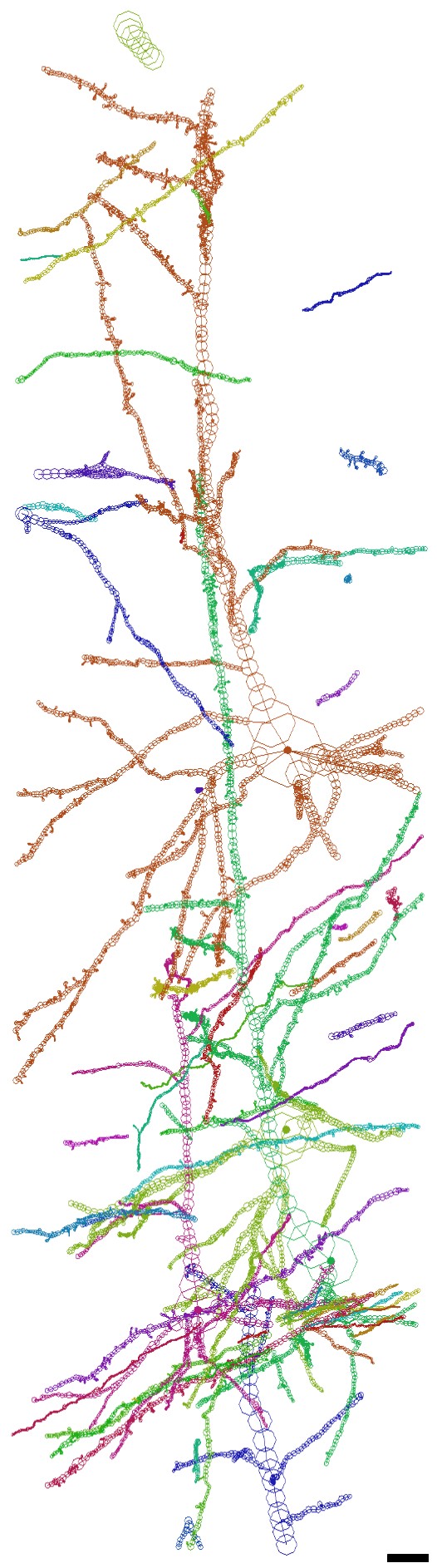

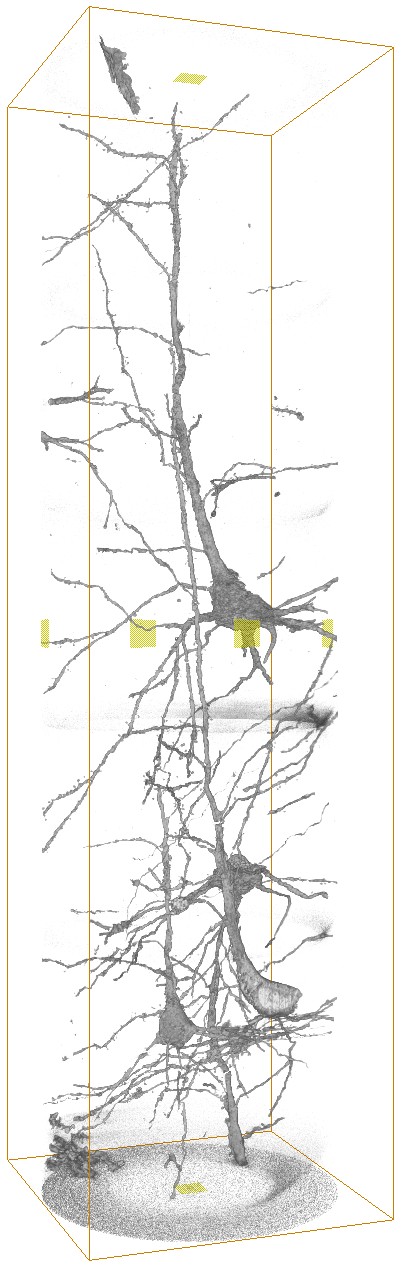


**Supplementary Figure 1 (cont'd).** Three-dimensional renderings of tissue structures and their Cartesian coordinate models. Scale bars: 10 μm. (**K**) Rendering of the N8A dataset of the schizophrenia N8 case. Voxel values from 80 to 800 are rendered. (**L**) Model of N8A.


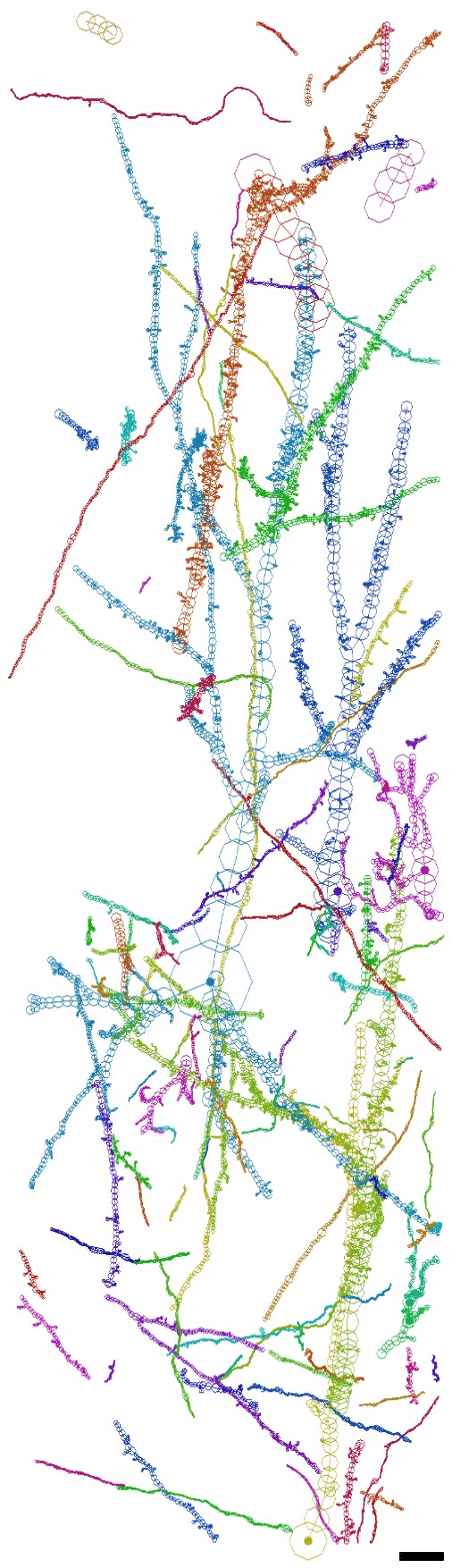

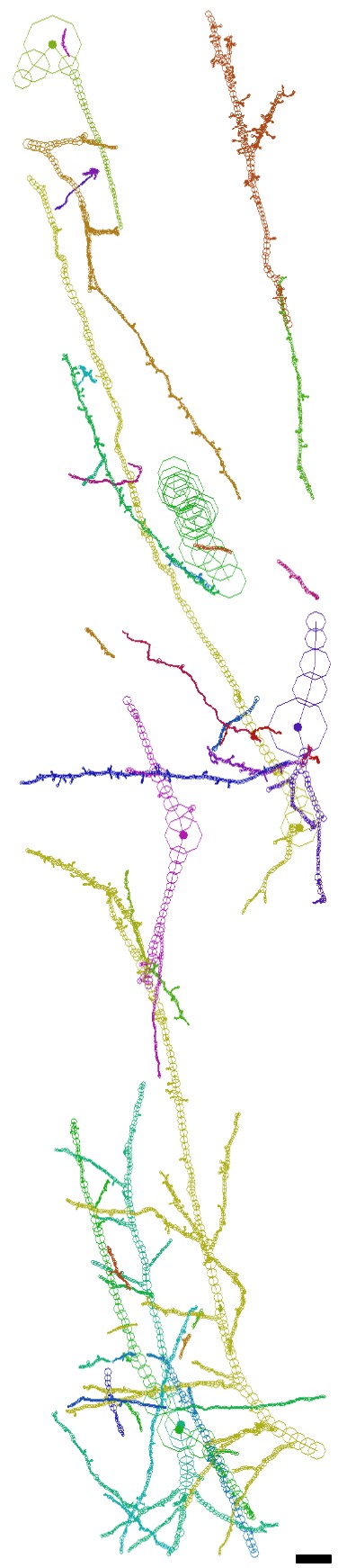


(**A**) S5B structure. (**B**) S5C structure.

**Supplementary Figure 2.** Cartesian coordinate models of schizophrenia case structures drawn with the MCTrace software. The pial surface is toward the top. Structural model constituents are color-coded. Nodes constituting the model are indicated with octagons and soma nodes with dots. Scale bars: 10 μm.


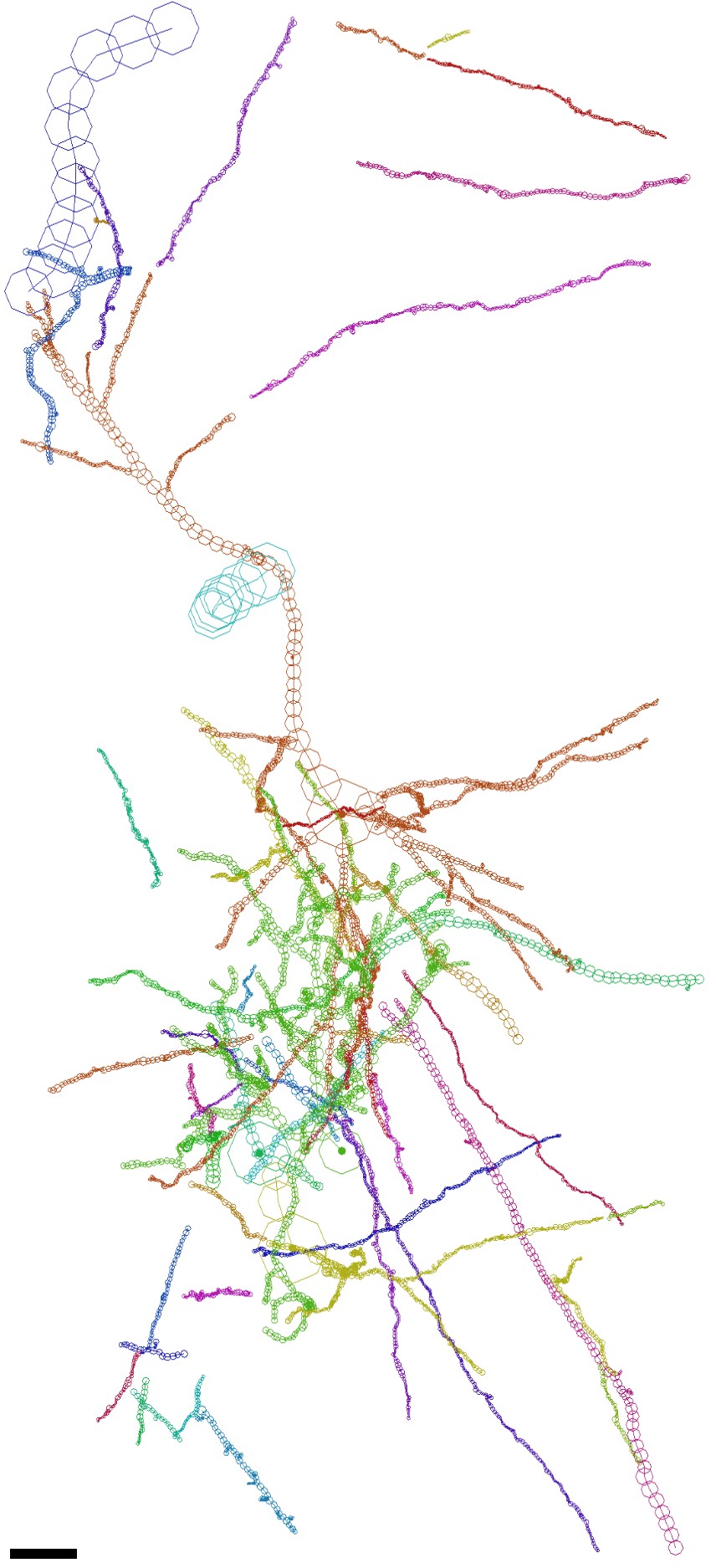

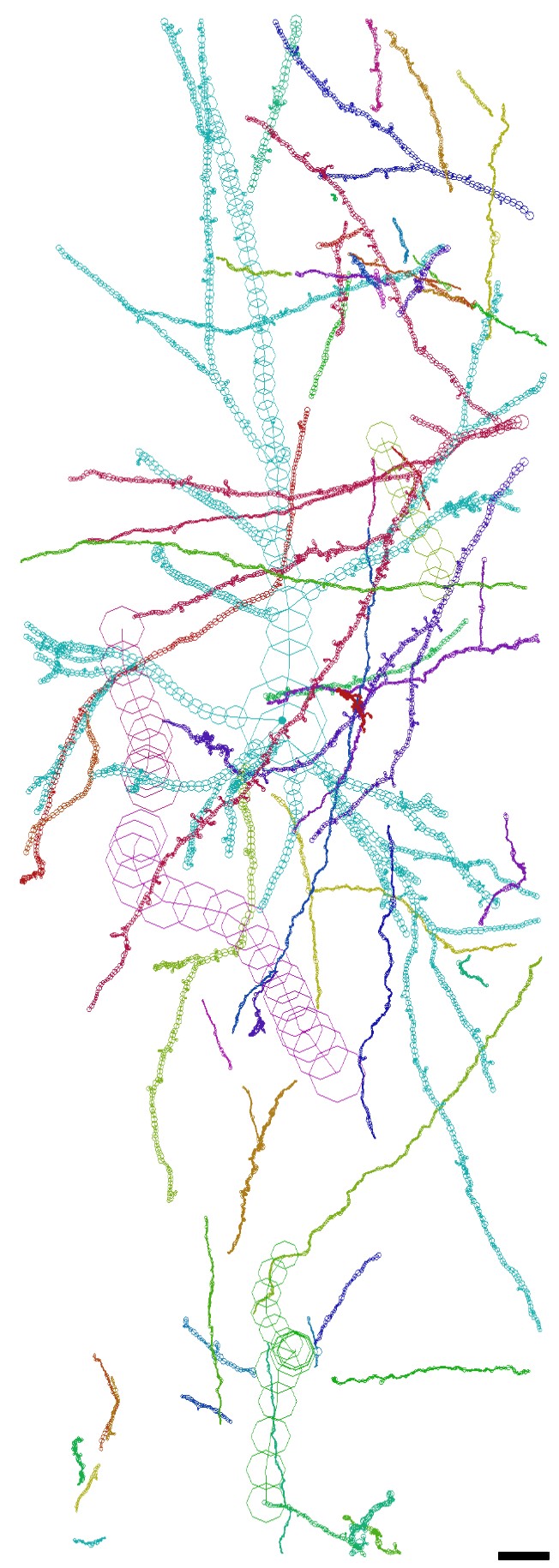


(**C**) S6B structure. (**D**) S6C structure.

**Supplementary Figure 2 (cont'd).** Cartesian coordinate models of schizophrenia case structures. The pial surface is toward the top. Nodes constituting the model are indicated with octagons and soma nodes with dots. Scale bars: 10 μm.


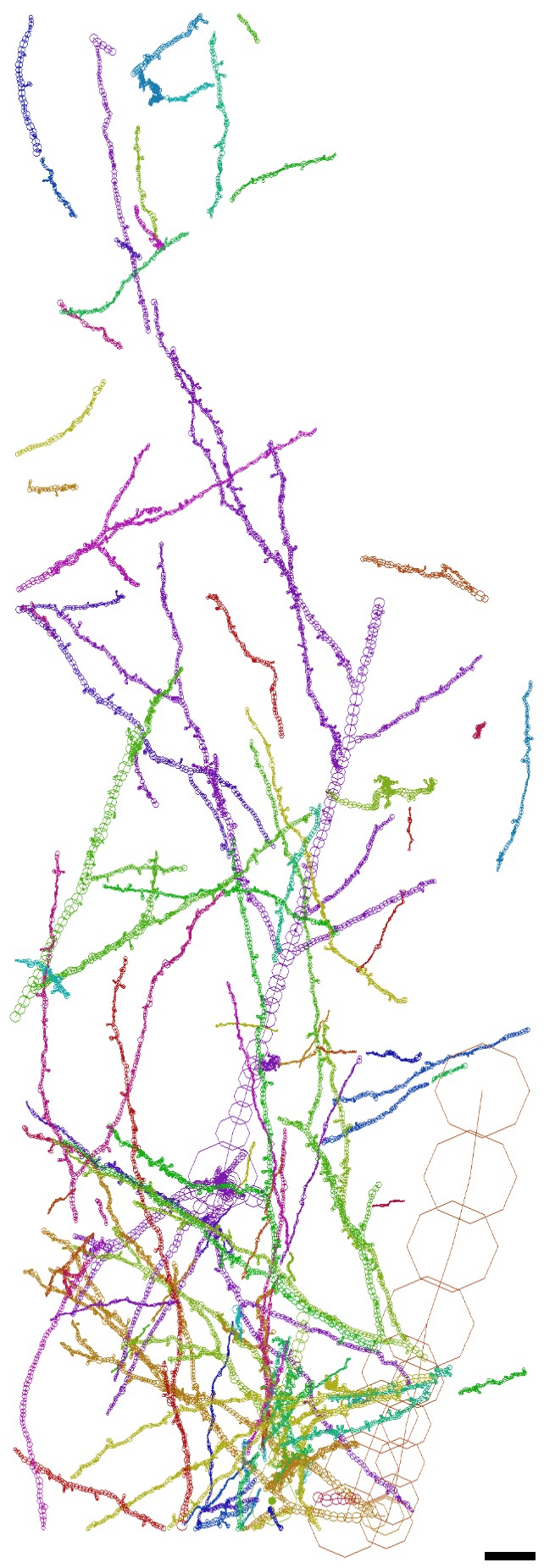


(**E**) S6D structure.

**Supplementary Figure 2 (cont'd).** Cartesian coordinate models of schizophrenia case structures. The pial surface is toward the top. Nodes constituting the model are indicated with octagons and soma nodes with dots. Scale bars: 10 μm.


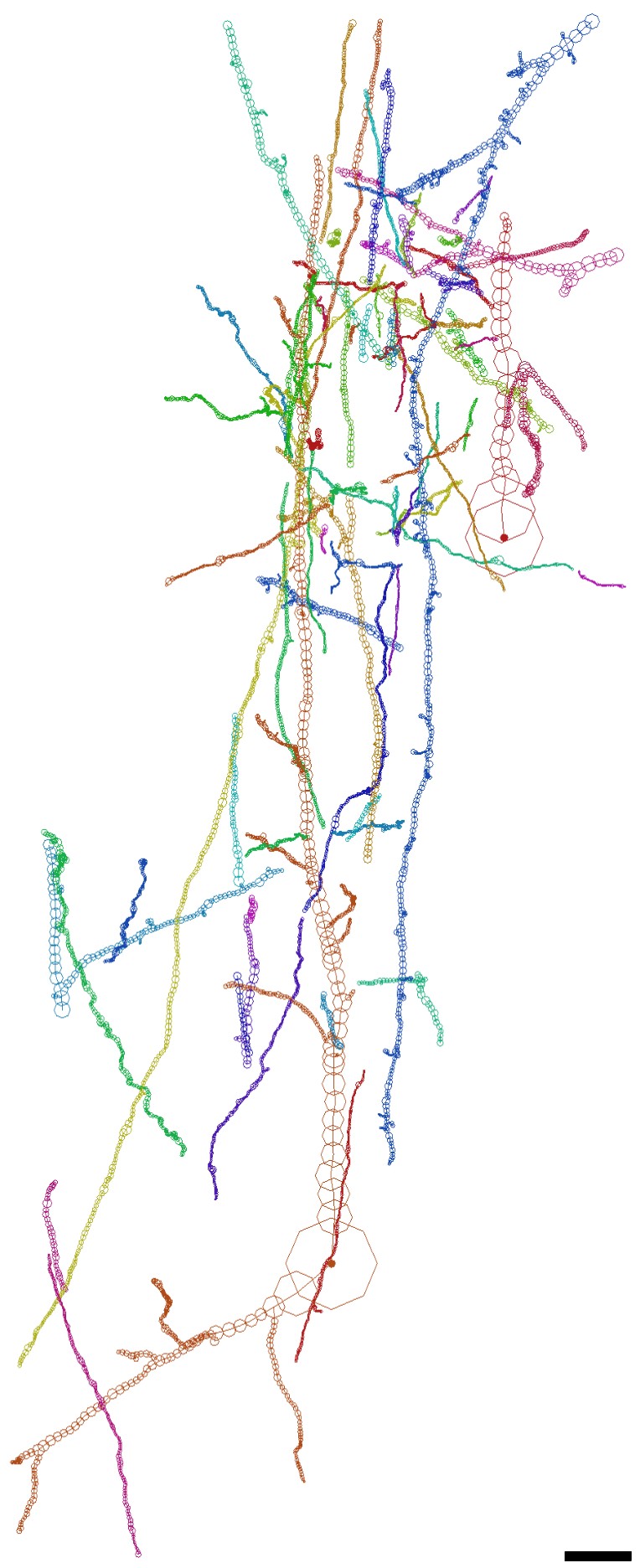

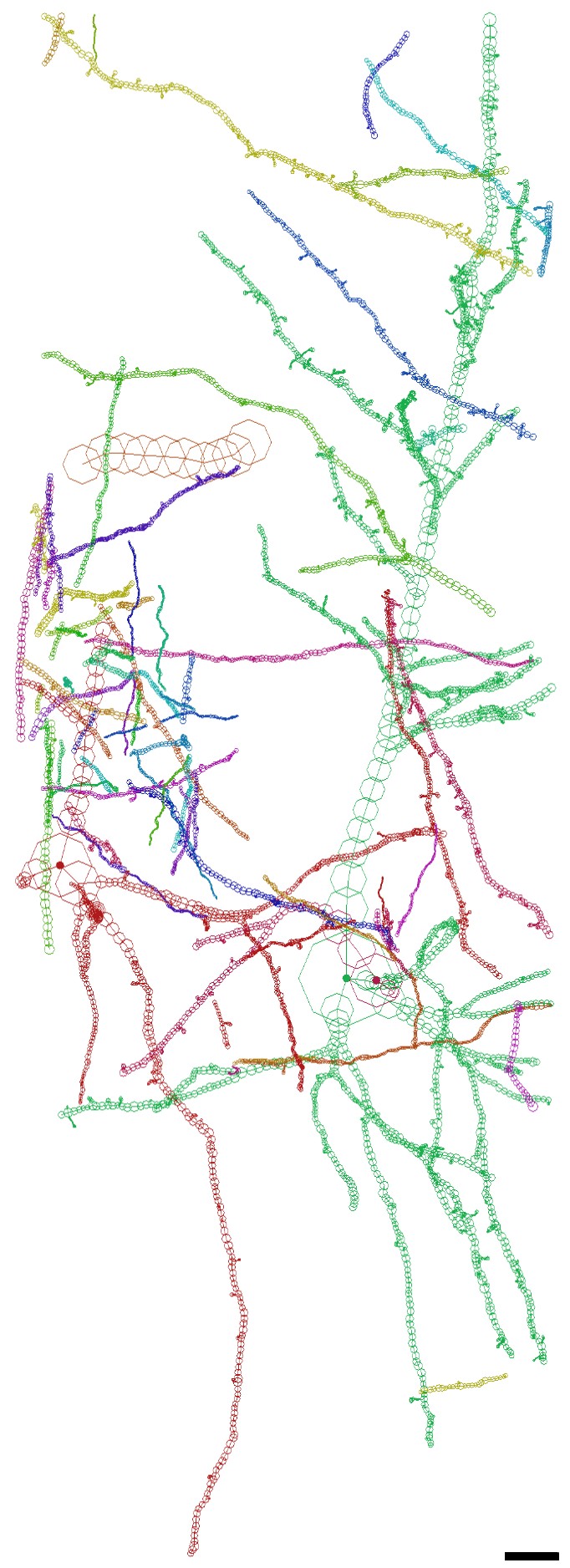


(**F**) S7B structure. (**G**) S7C structure.

**Supplementary Figure 2 (cont'd).** Cartesian coordinate models of schizophrenia case structures. The pial surface is toward the top. Nodes constituting the model are indicated with octagons and soma nodes with dots. Scale bars: 10 μm.


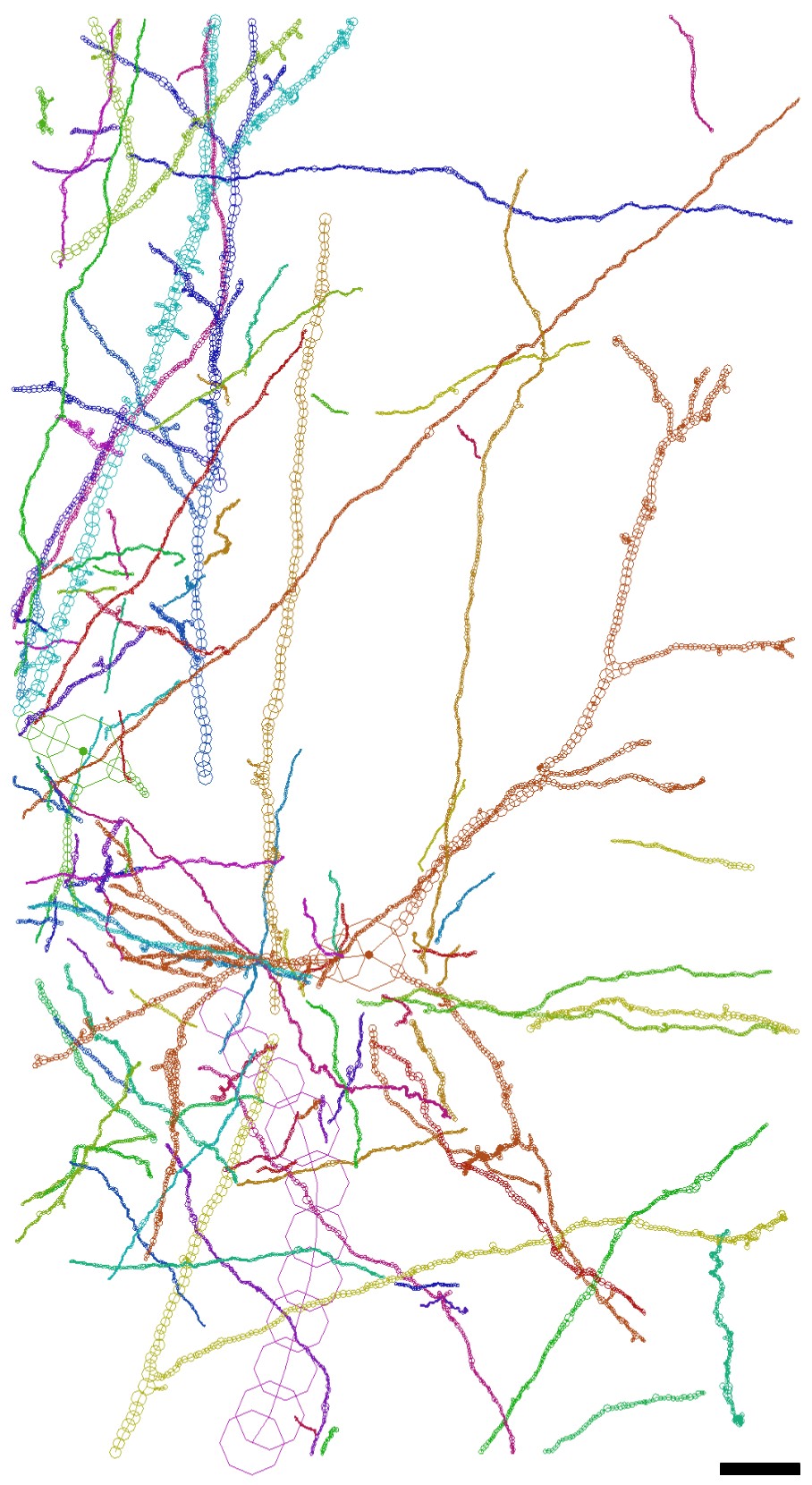

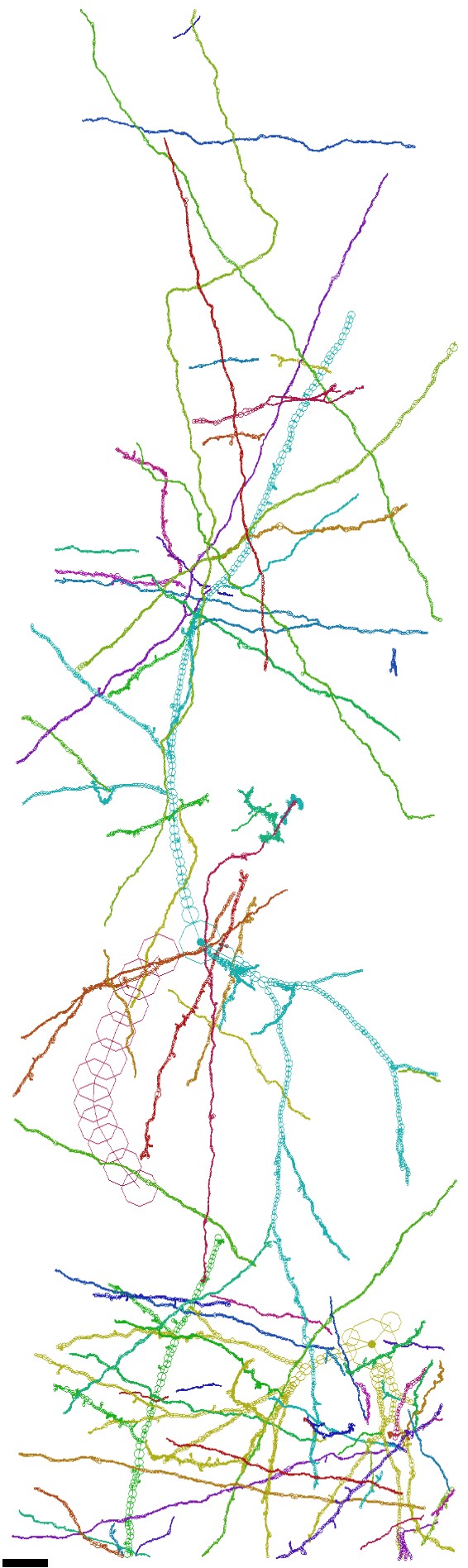


(**H**) S8B structure. (**I**) S8C structure.

**Supplementary Figure 2 (cont'd).** Cartesian coordinate models of schizophrenia case structures. The pial surface is toward the top. Nodes constituting the model are indicated with octagons and soma nodes with dots. Scale bars: 10 μm.


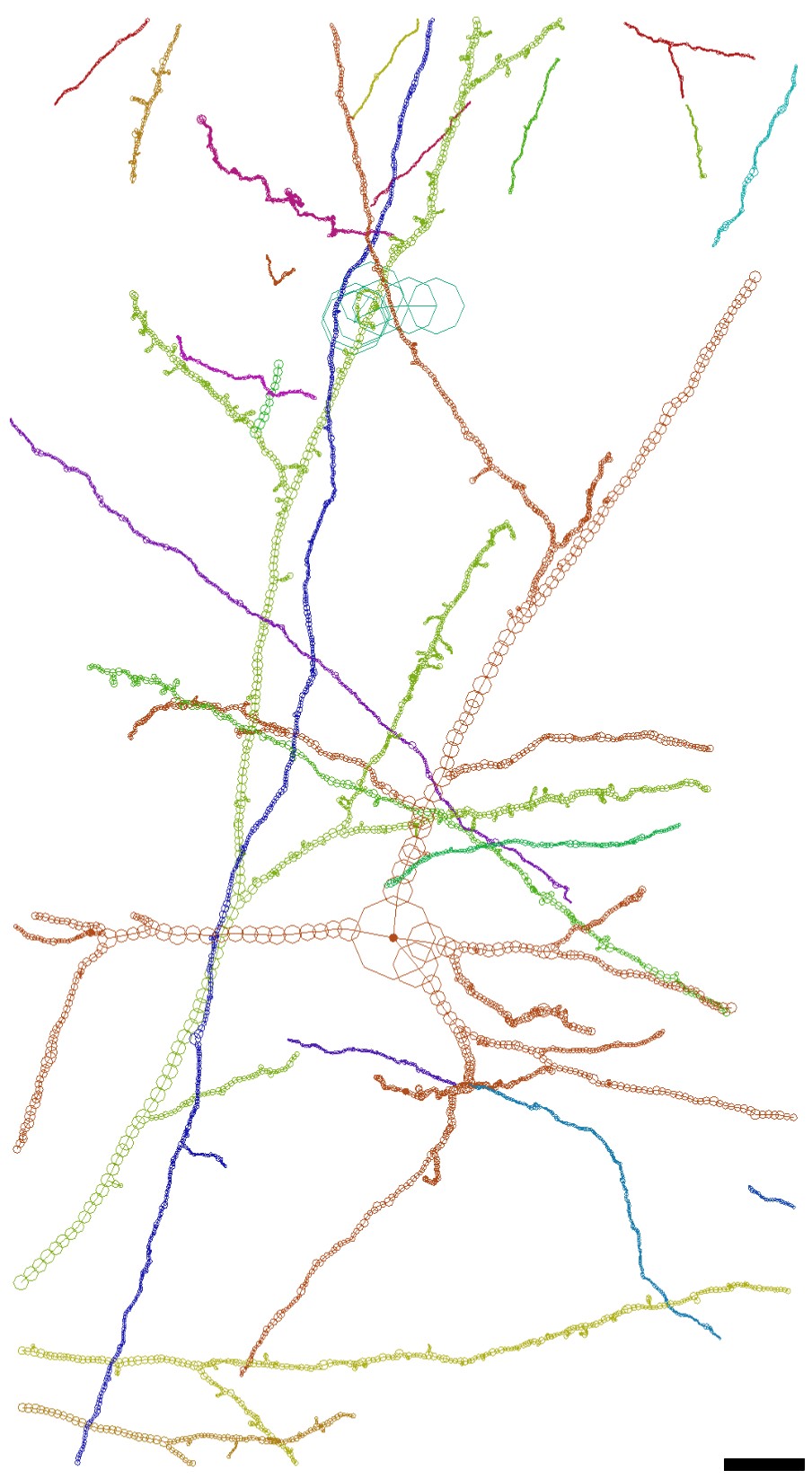

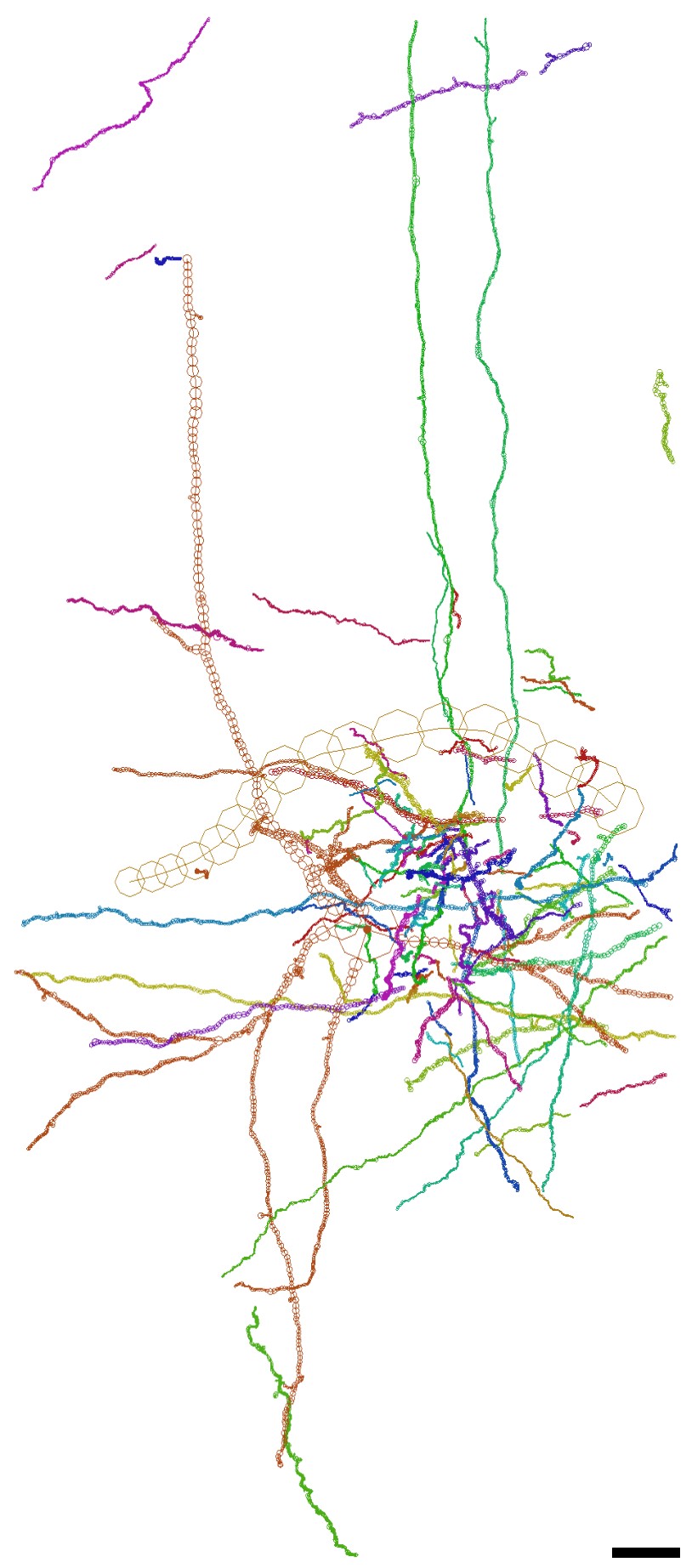


(**J**) S8D structure. (**K**) S8E structure.

**Supplementary Figure 2 (cont'd).** Cartesian coordinate models of schizophrenia case structures. The pial surface is toward the top. Nodes constituting the model are indicated with octagons and soma nodes with dots. Scale bars: 10 μm.


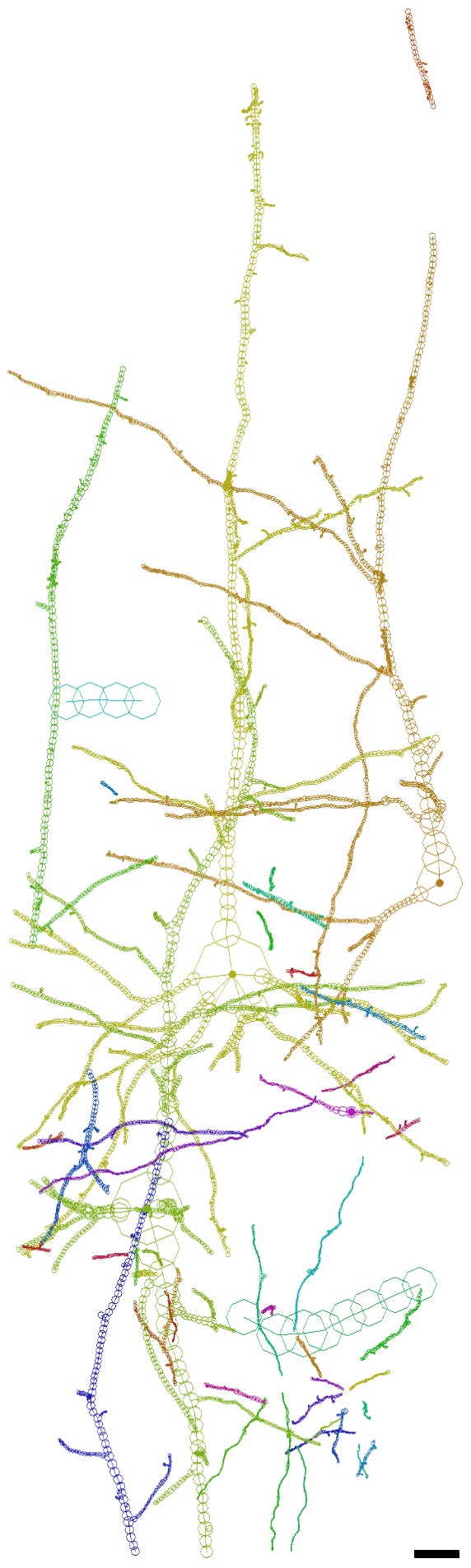

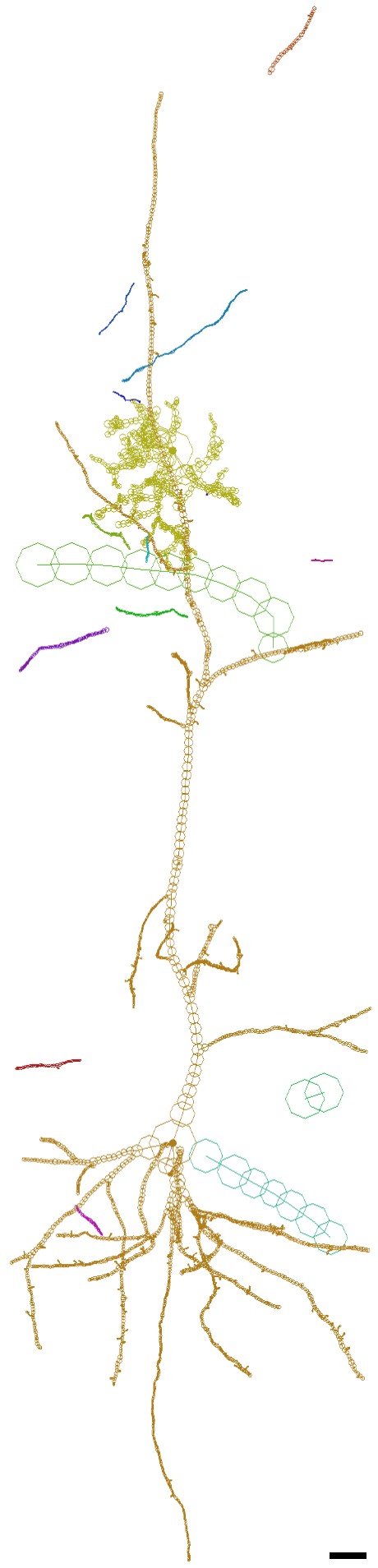


(**A**) N5B structure. (**B**) N5C structure.

**Supplementary Figure 3.** Cartesian coordinate models of control case structures drawn with the MCTrace software. The pial surface is toward the top. Structural model constituents are color-coded. Nodes constituting the model are indicated with octagons and soma nodes with dots. Scale bars: 10 μm.


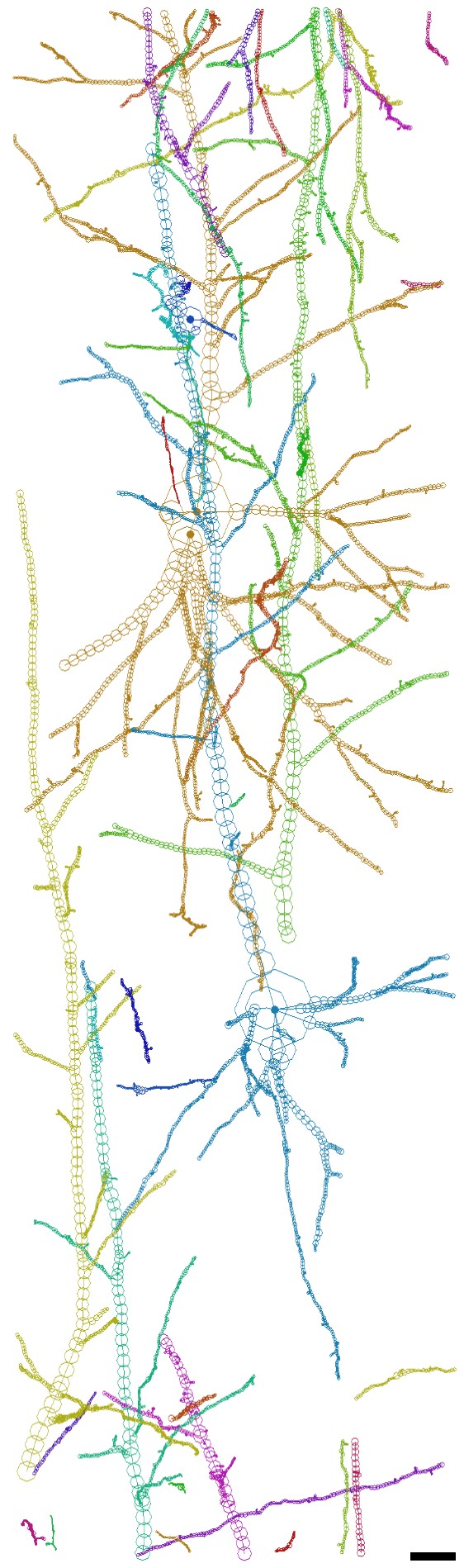

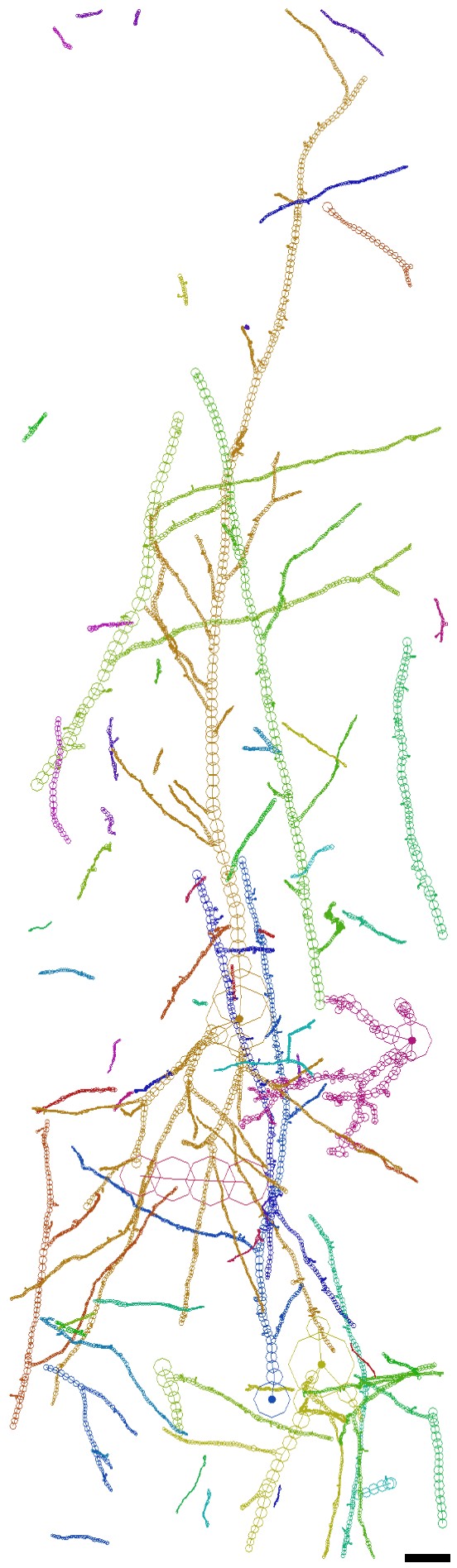


(**C**) N5D structure. (**D**) N5E structure.

**Supplementary Figure 3 (cont'd).** Cartesian coordinate models of control case structures. The pial surface is toward the top. Nodes constituting the model are indicated with octagons and soma nodes with dots. Scale bars: 10 μm.


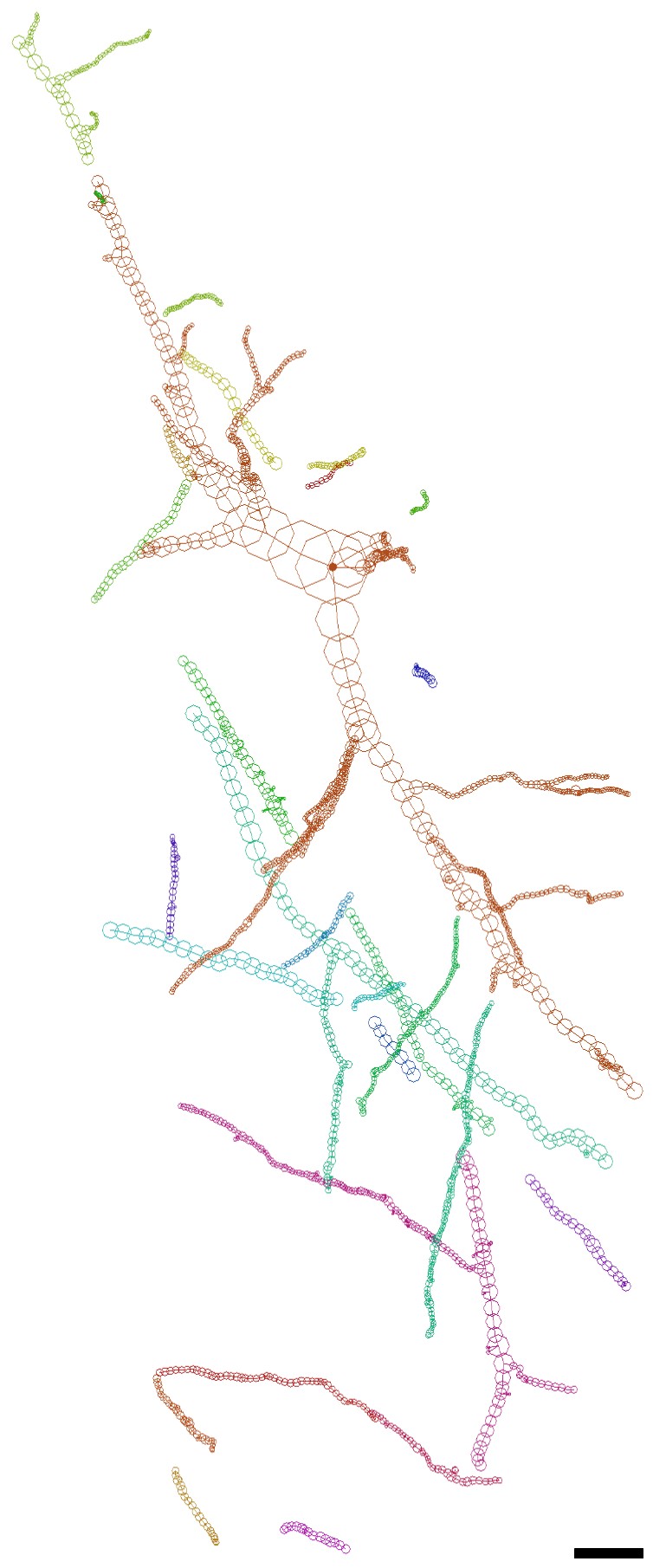

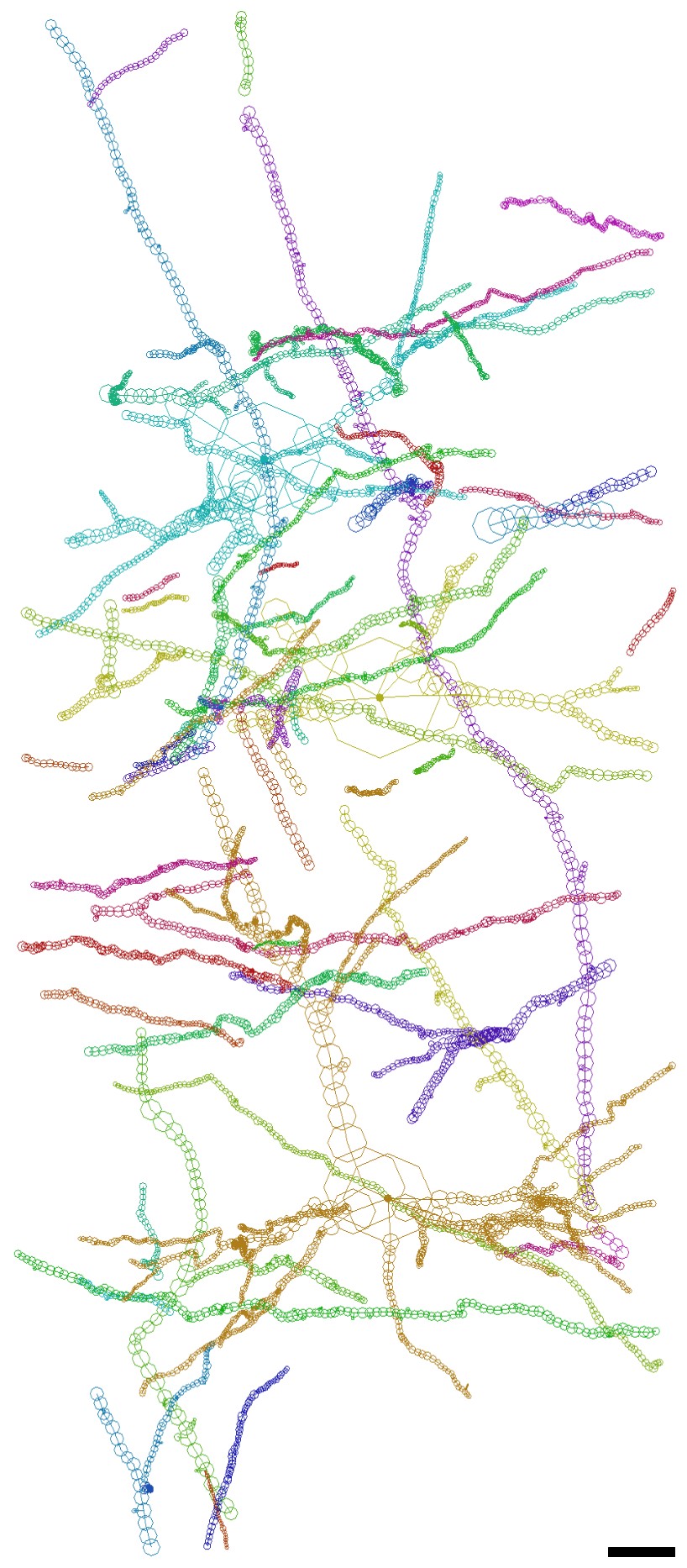


(**E**) N6B structure. (**F**) N6C structure.

**Supplementary Figure 3 (cont'd).** Cartesian coordinate models of control case structures. The pial surface is toward the top. Nodes constituting the model are indicated with octagons and soma nodes with dots. Scale bars: 10 μm.


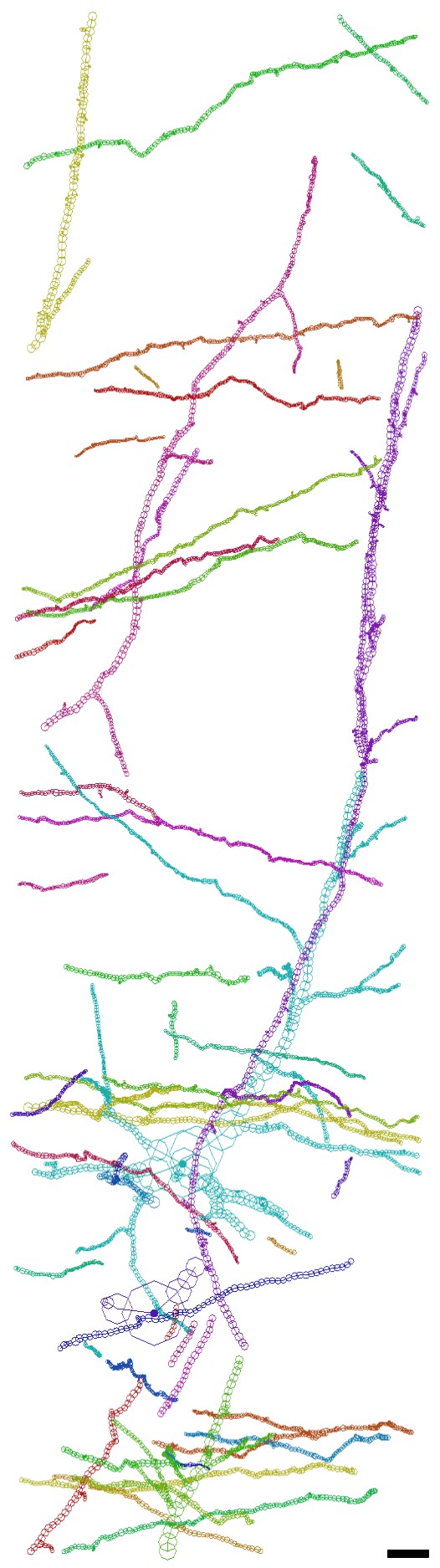

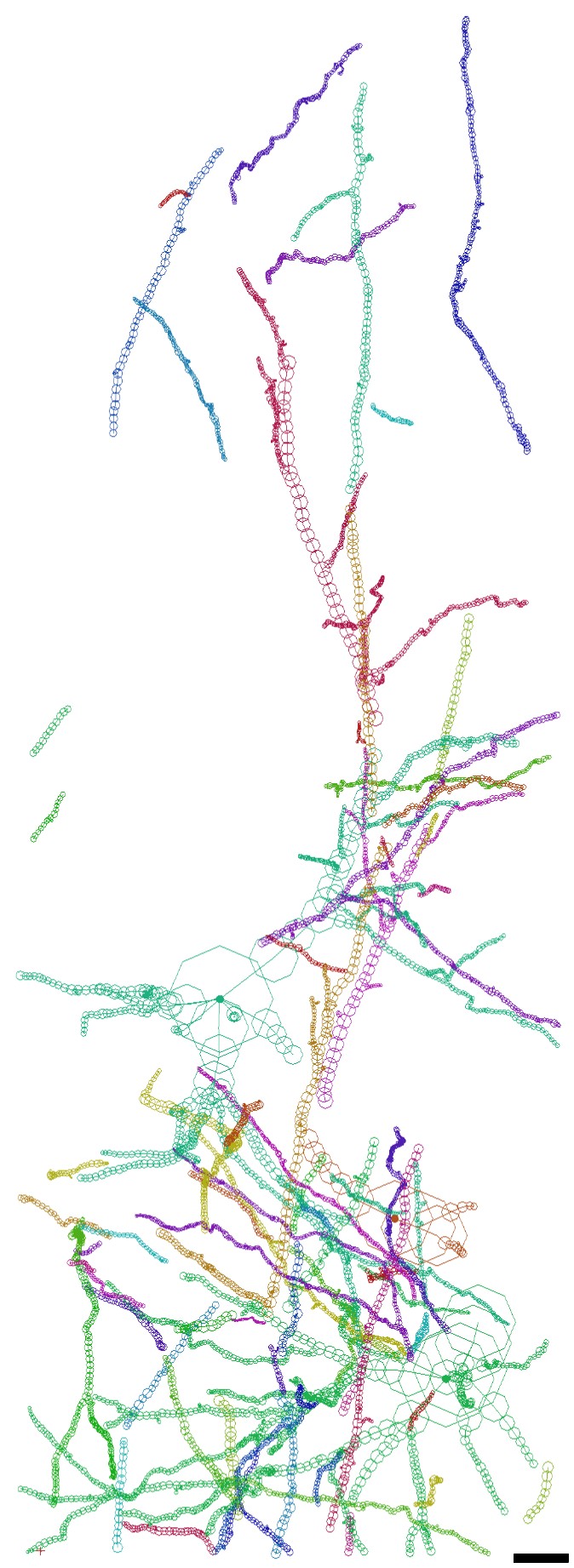


(**G**) N6D structure. (**H**) N6E structure.

**Supplementary Figure 3 (cont'd).** Cartesian coordinate models of control case structures. The pial surface is toward the top. Nodes constituting the model are indicated with octagons and soma nodes with dots. Scale bars: 10 μm.


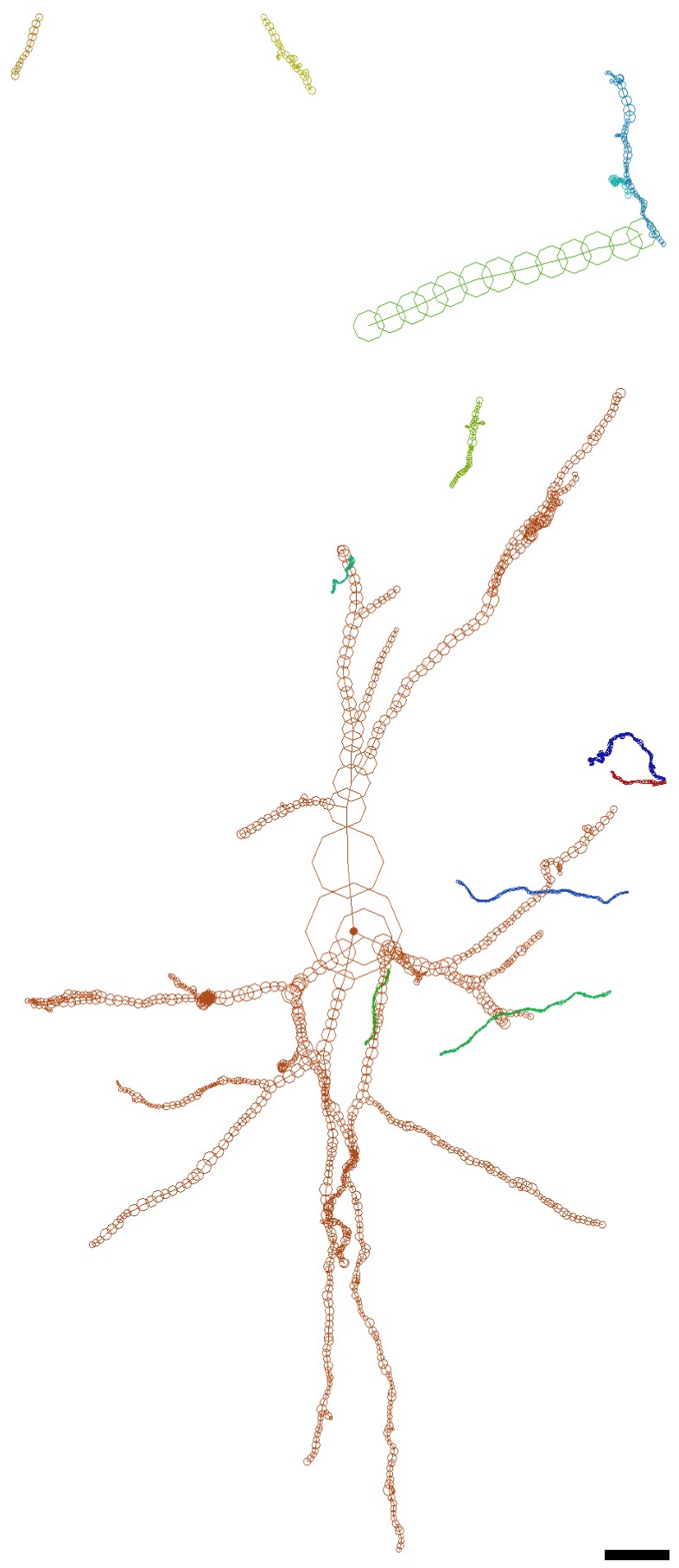

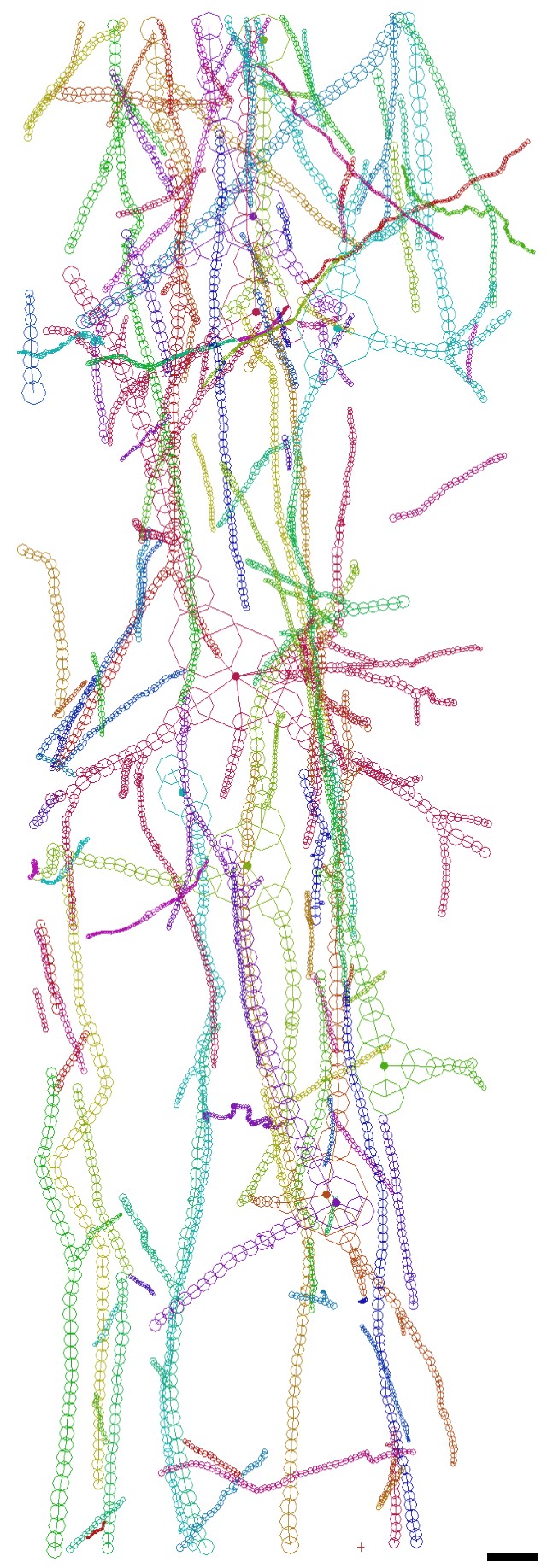


(**I**) N7B structure. (**J**) N7C structure.

**Supplementary Figure 3 (cont'd).** Cartesian coordinate models of control case structures. The pial surface is toward the top. Nodes constituting the model are indicated with octagons and soma nodes with dots. Scale bars: 10 μm.


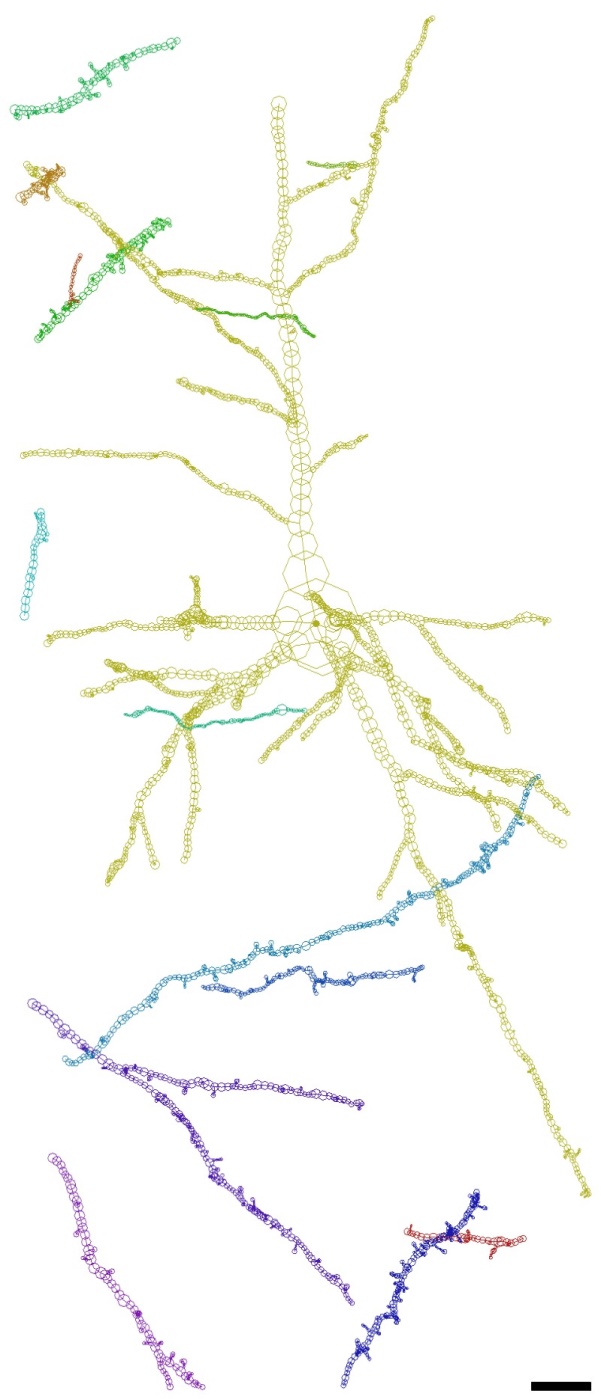


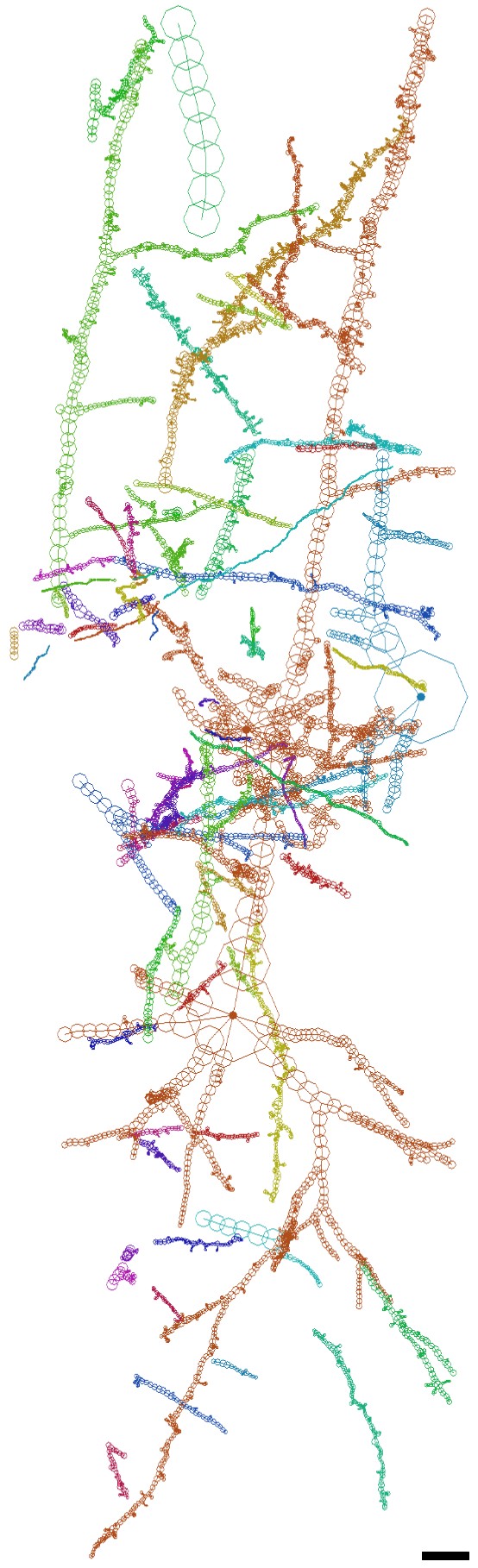


(**K**) N8B structure. (**L**) N8C structure.

**Supplementary Figure 3 (cont'd).** Cartesian coordinate models of control case structures. The pial surface is toward the top. Nodes constituting the model are indicated with octagons and soma nodes with dots. Scale bars: 10 μm.


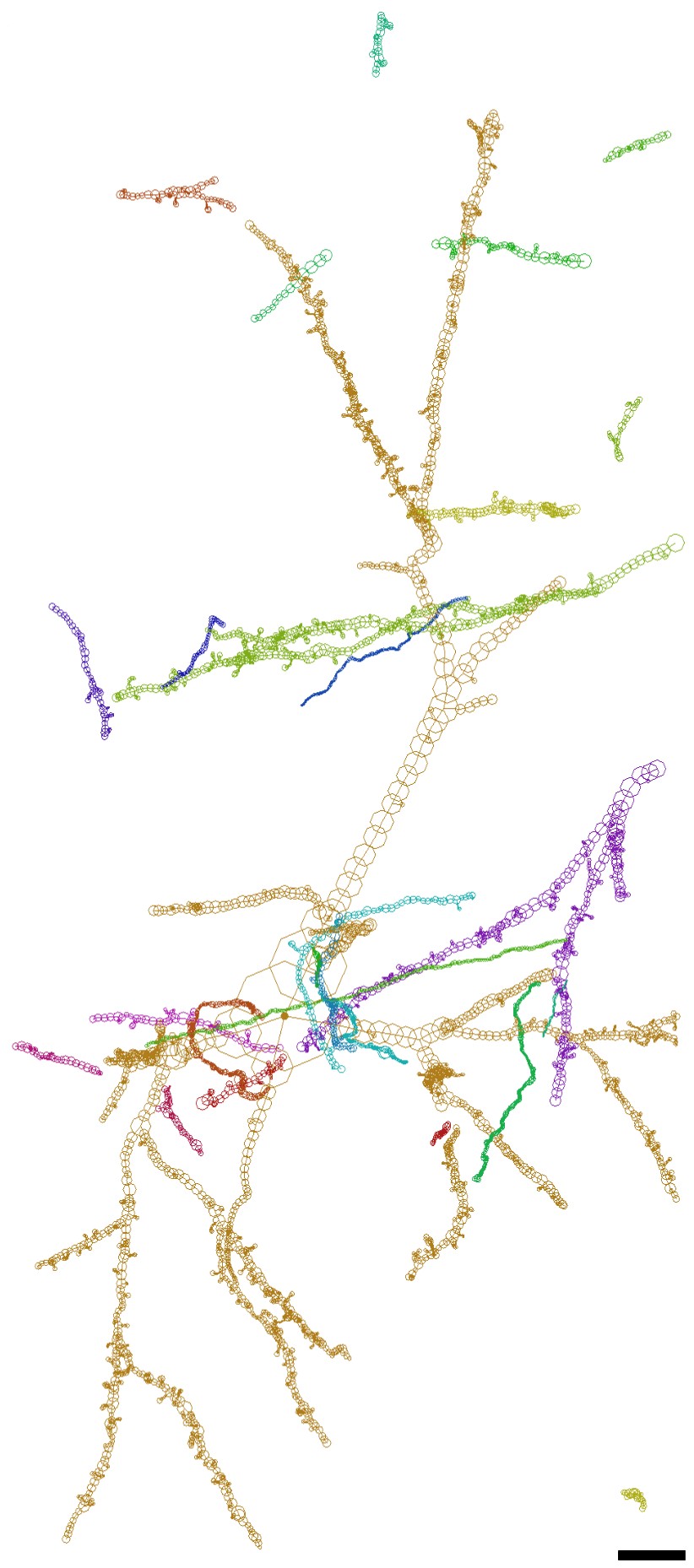

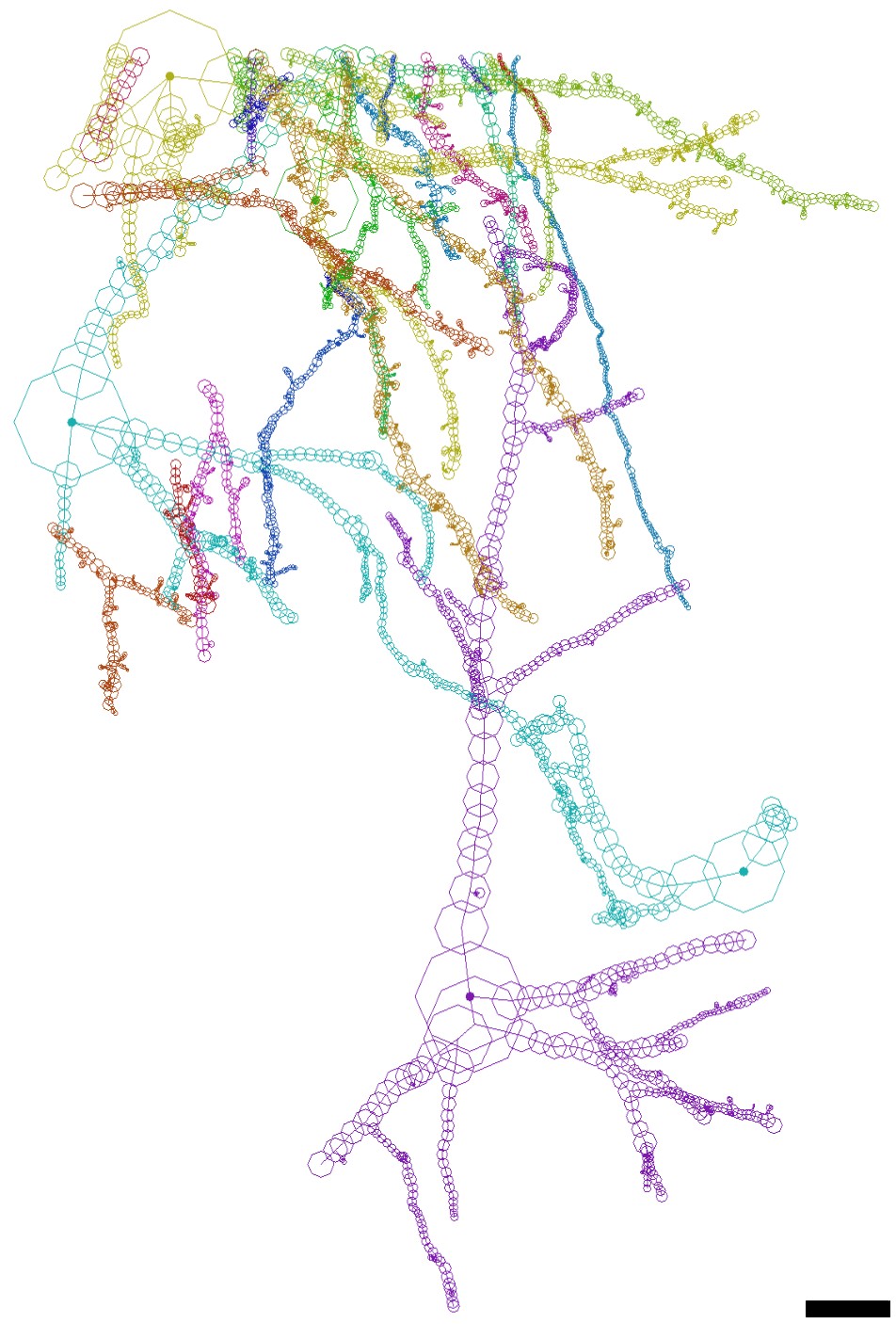


(**M**) N8D structure. (**N**) N8E structure.

**Supplementary Figure 3 (cont'd).** Cartesian coordinate models of control case structures. The pial surface is toward the top. Nodes constituting the model are indicated with octagons and soma nodes with dots. Scale bars: 10 μm.

(A)


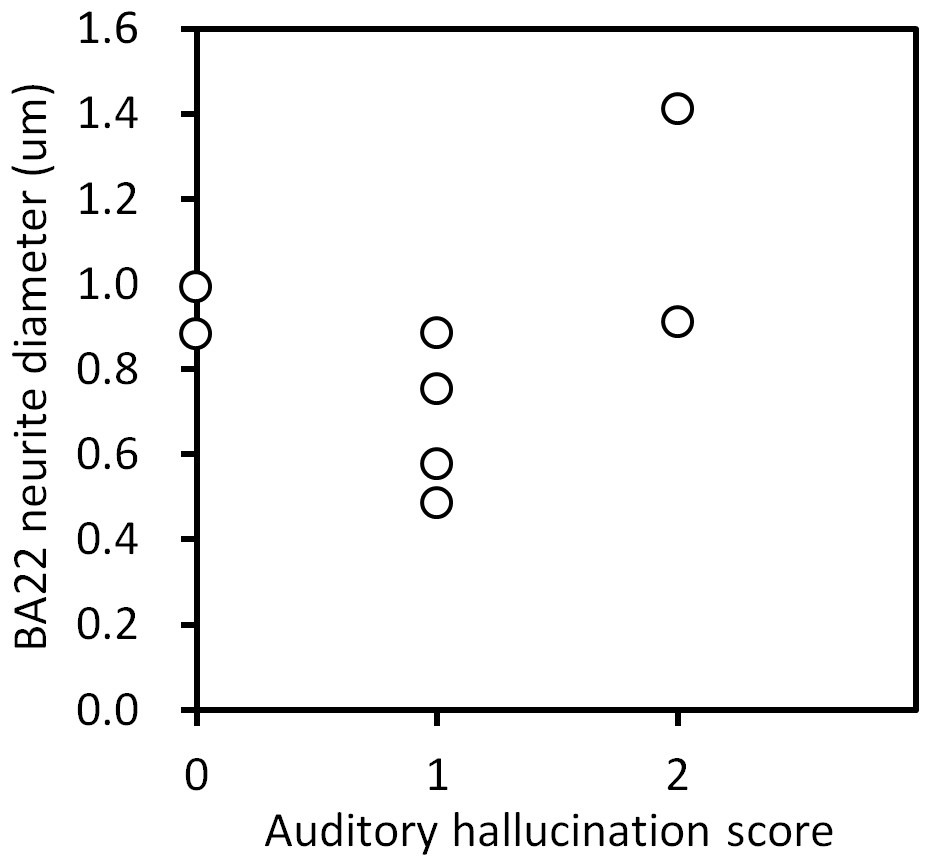


(B) (C)


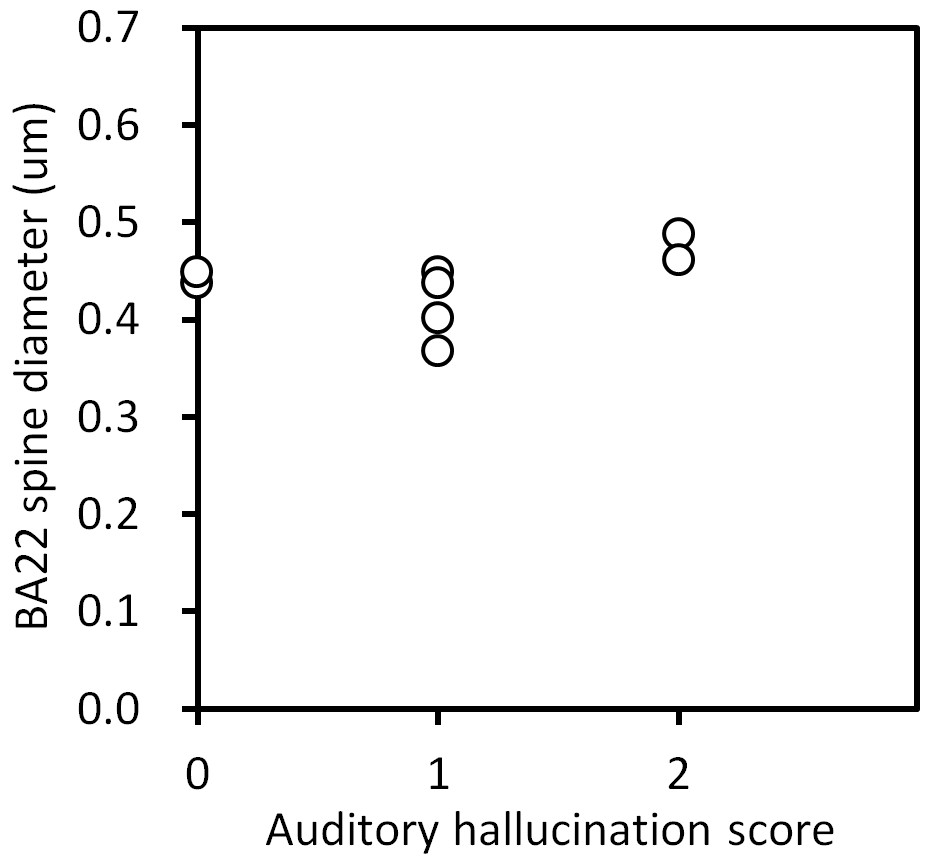

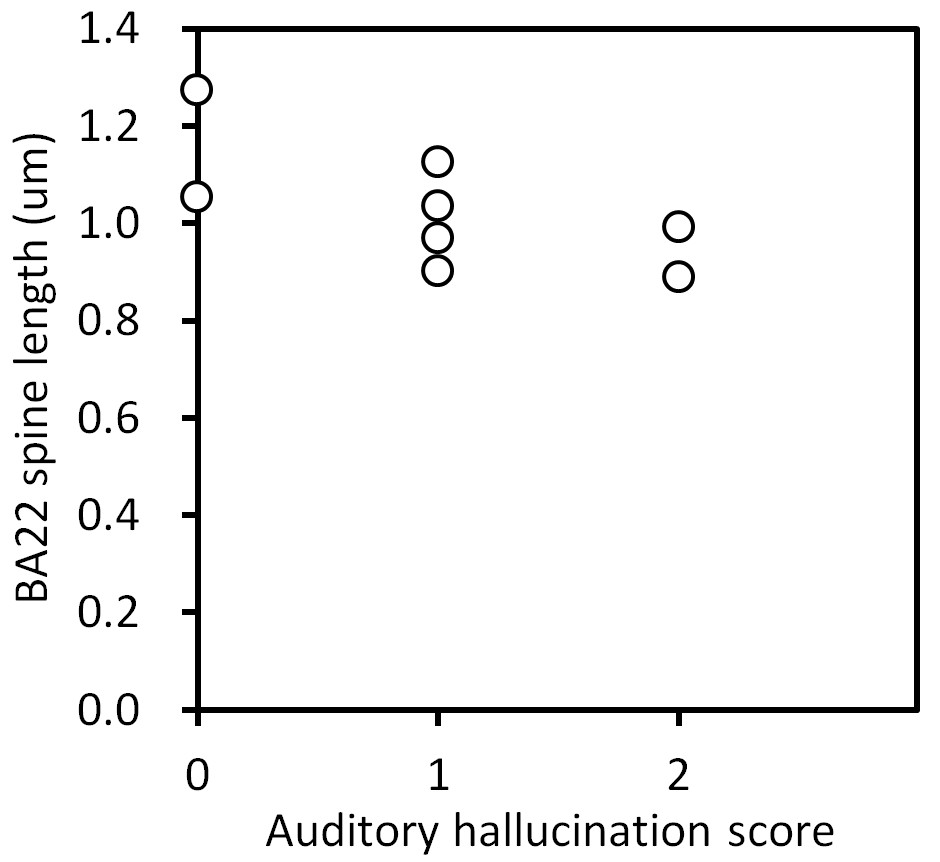


(D) (E)


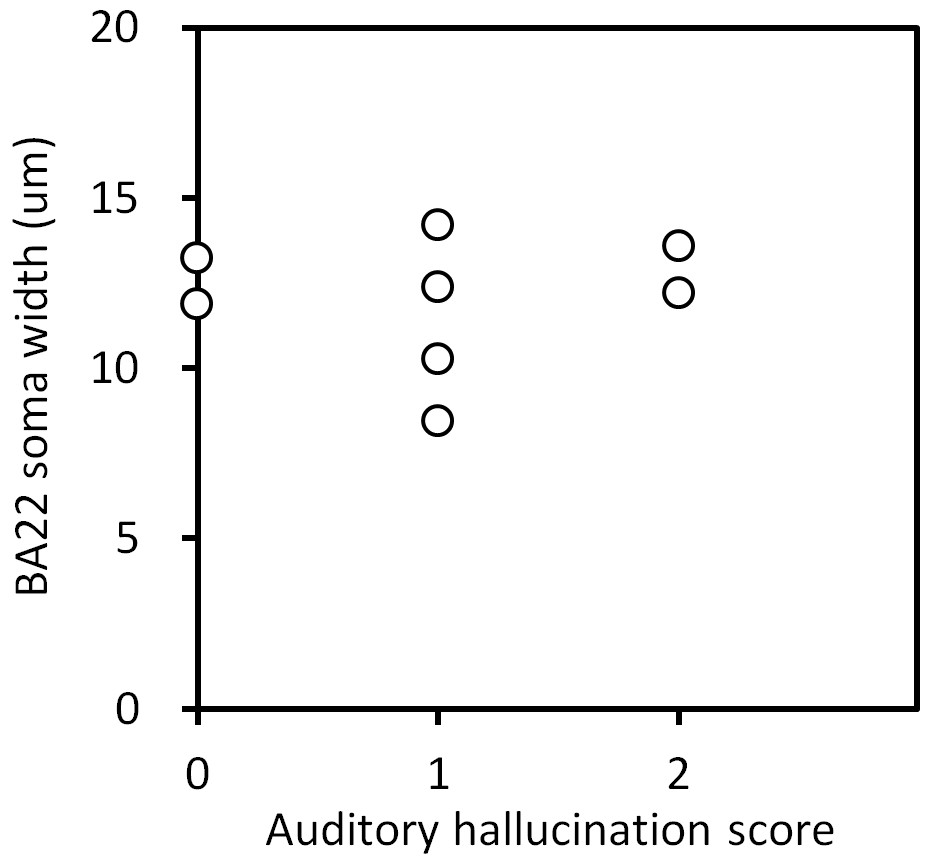

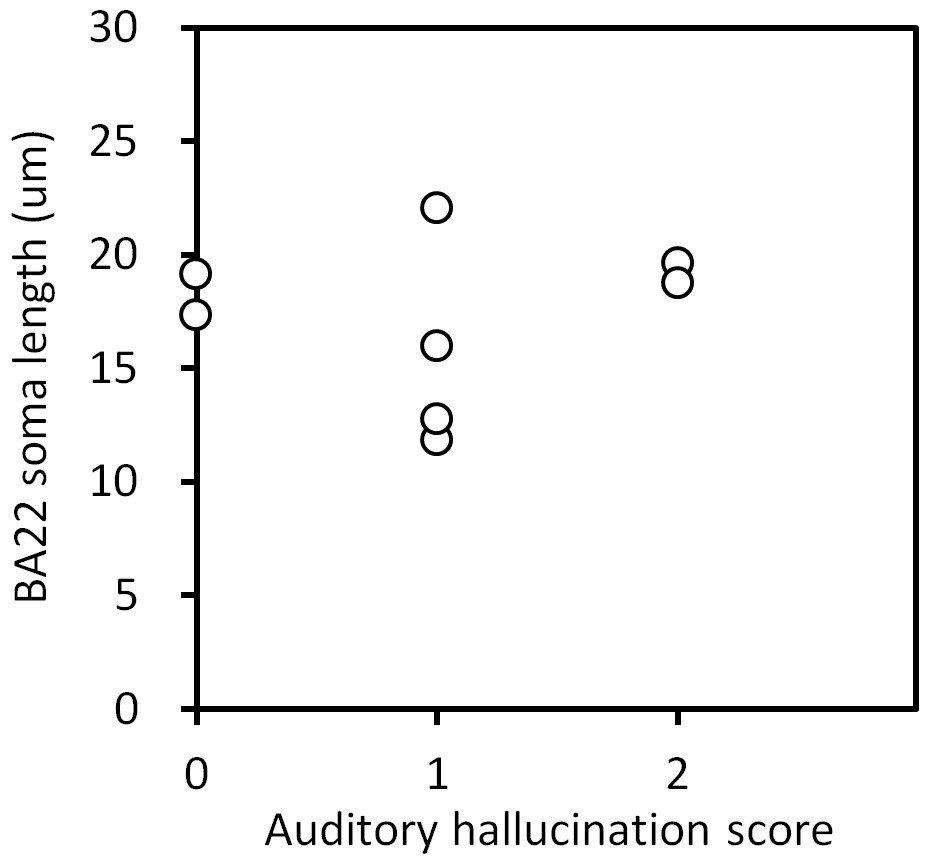


**Supplementary Figure 4.** Scatter plots of BA22 parameter of schizophrenia cases versus their auditory hallucination score. (A) Neurite diameter. (B) Spine diameter. (C) Spine length. (D) Soma width. (E) Soma length.

(A) (B) (C)


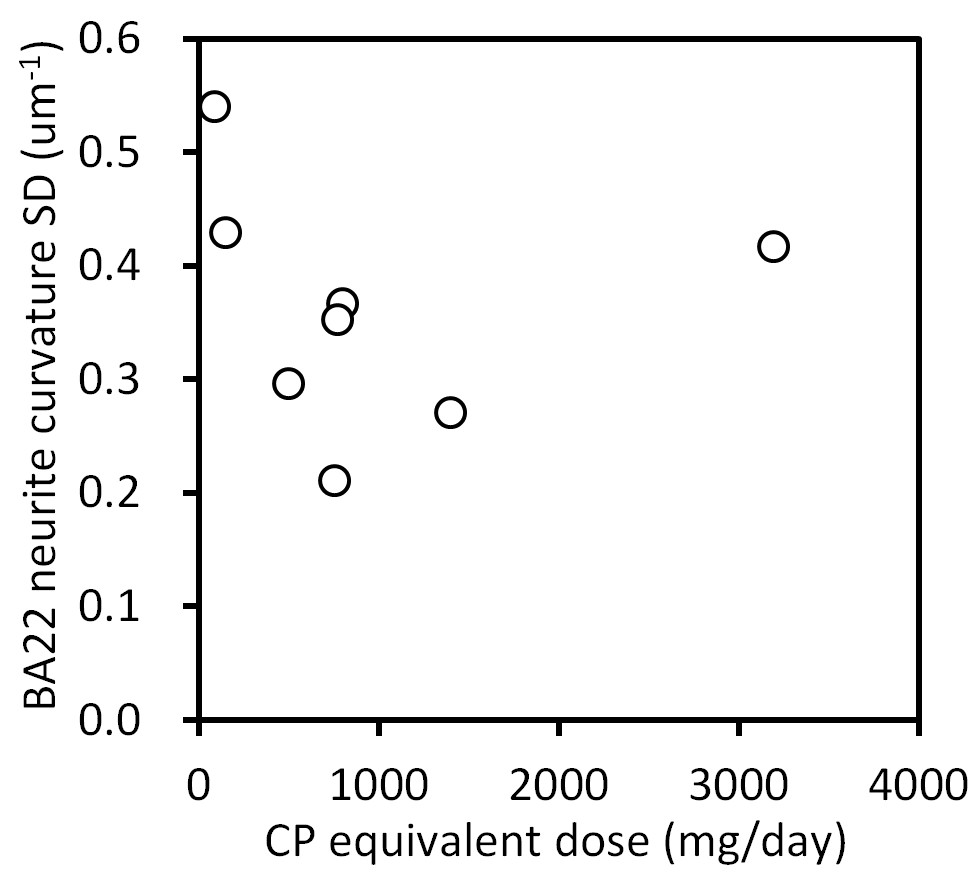

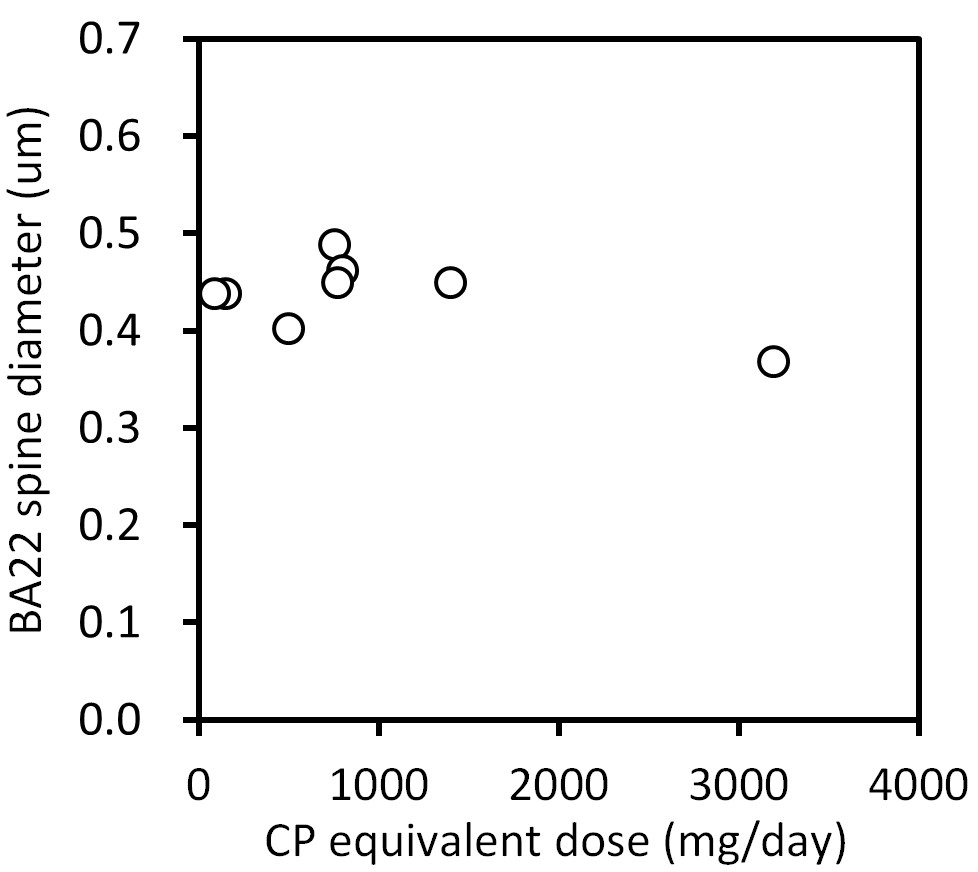

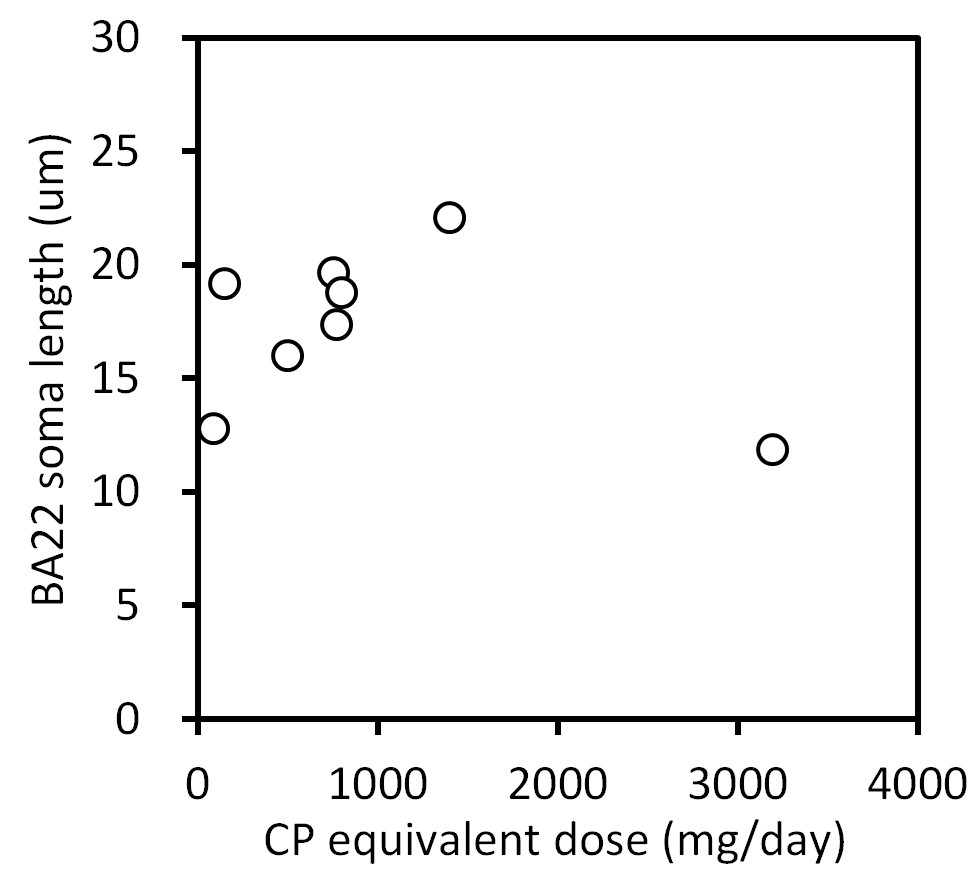


(D) (E) (F)


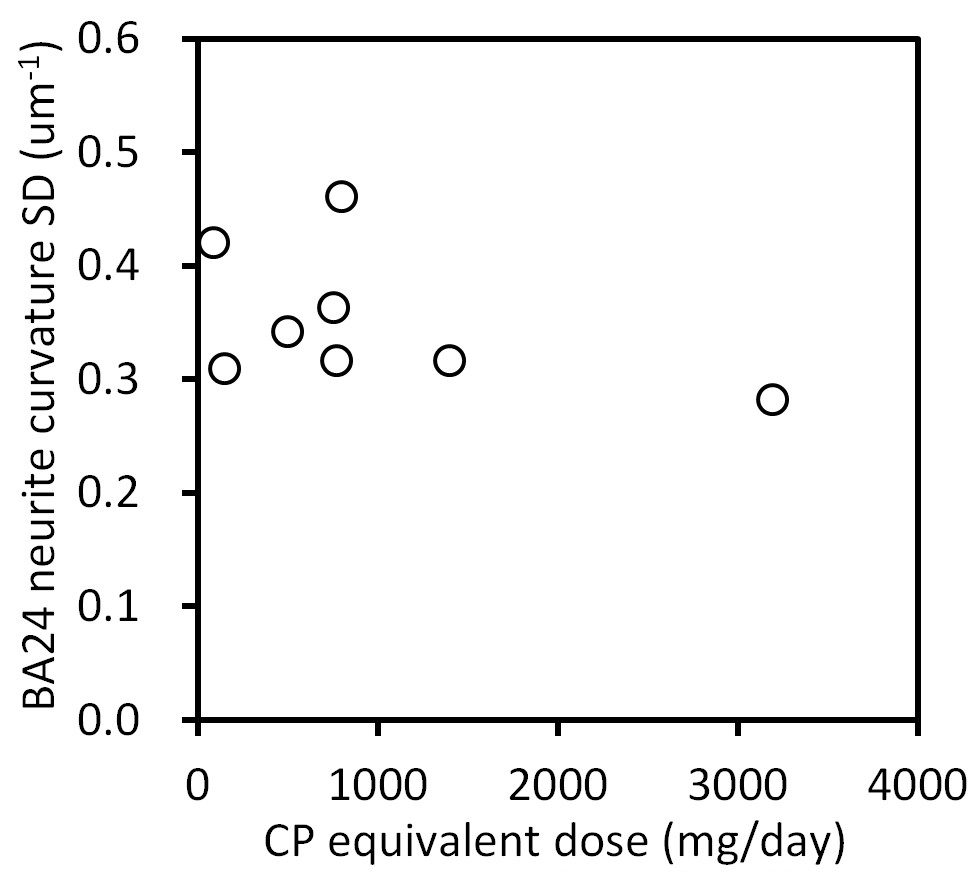

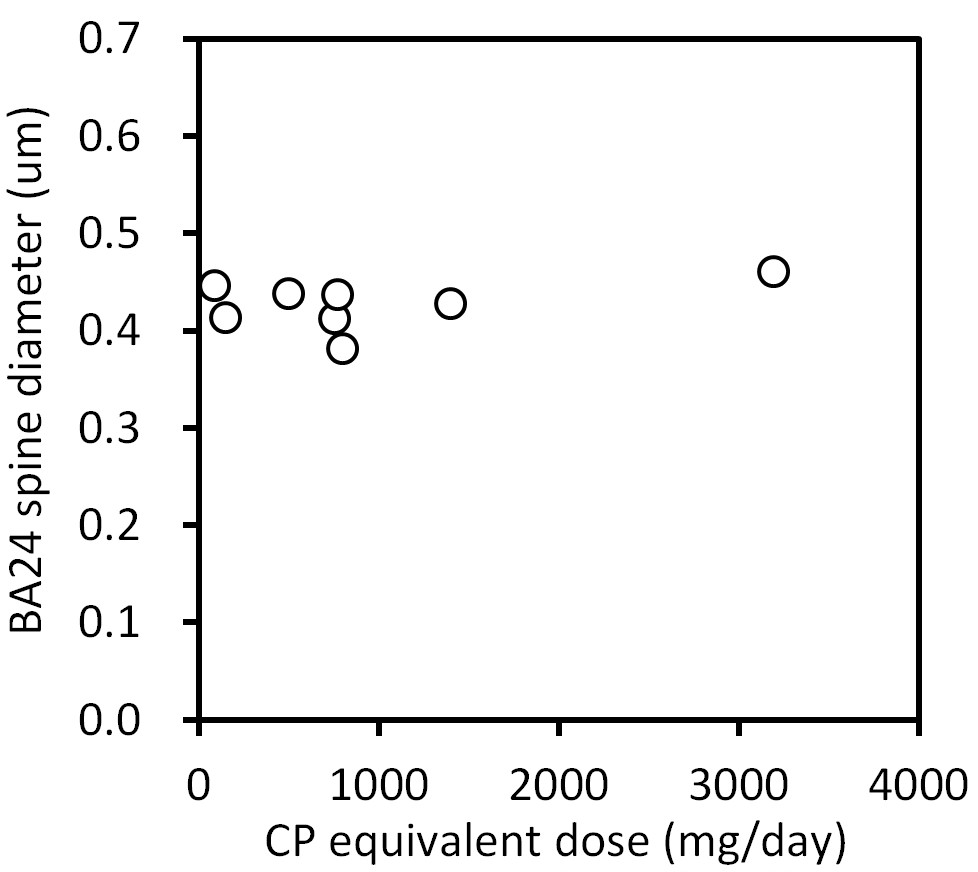

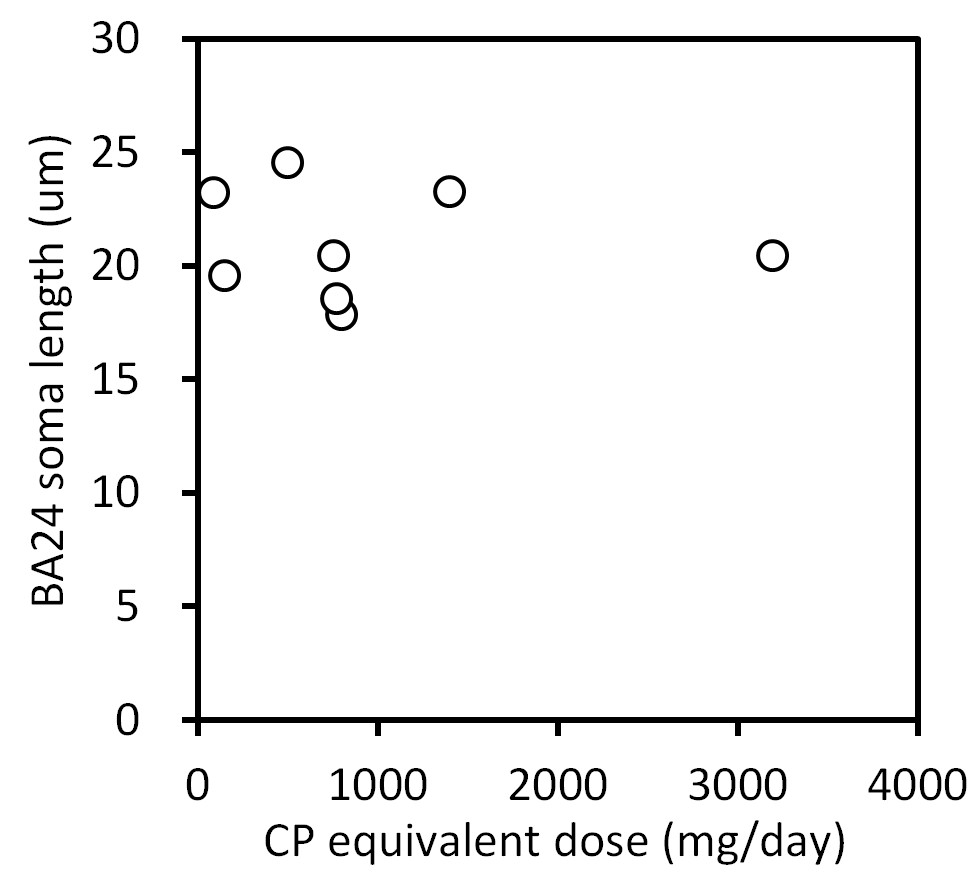


**Supplementary Figure 5.** Scatter plots of structural parameters of schizophrenia cases versus chlorpromazine (CP) equivalent daily dose. (A) Standard deviation of neurite curvatures of BA22. (B) Spine diameter of BA22. (C) Soma length of BA22. (D) Standard deviation of neurite curvature of BA24. (E) Spine diameter of BA24. (F) Soma length of BA24.

(A) (B)

**
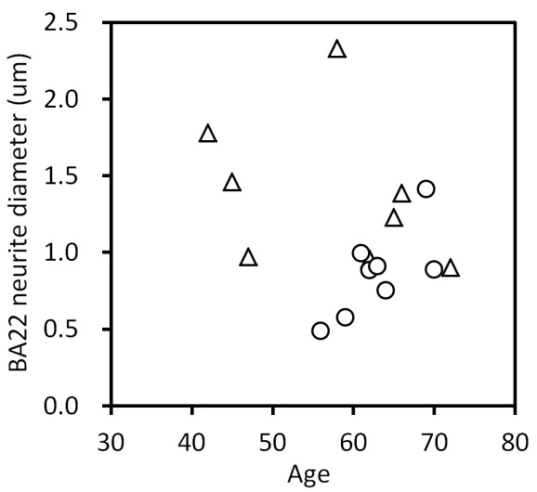

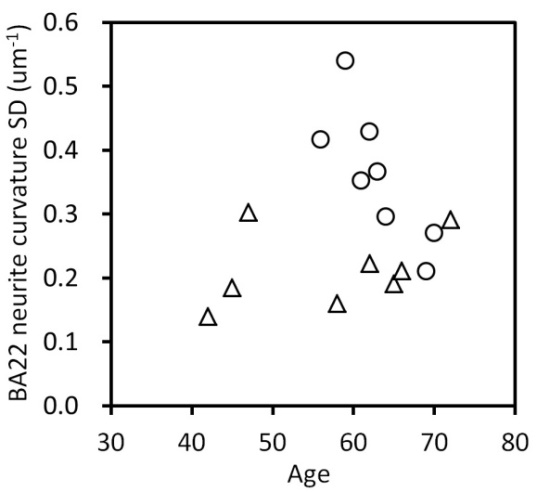
**

(C) (D)


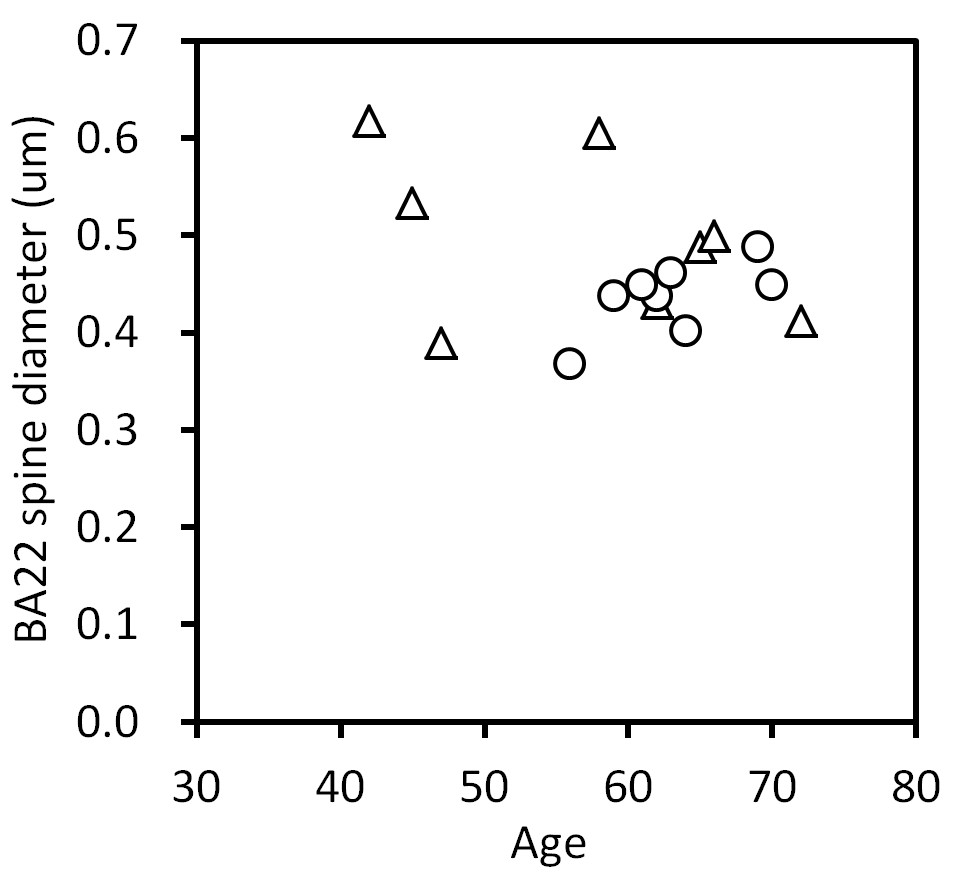

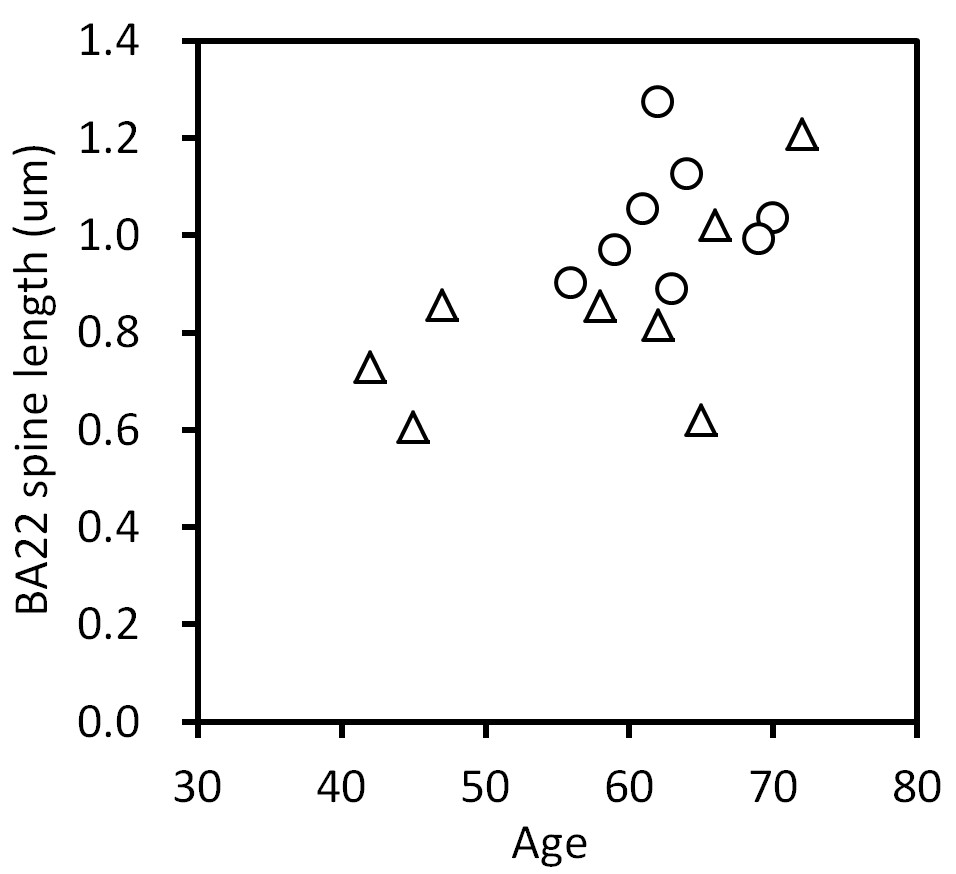


(E) (F)


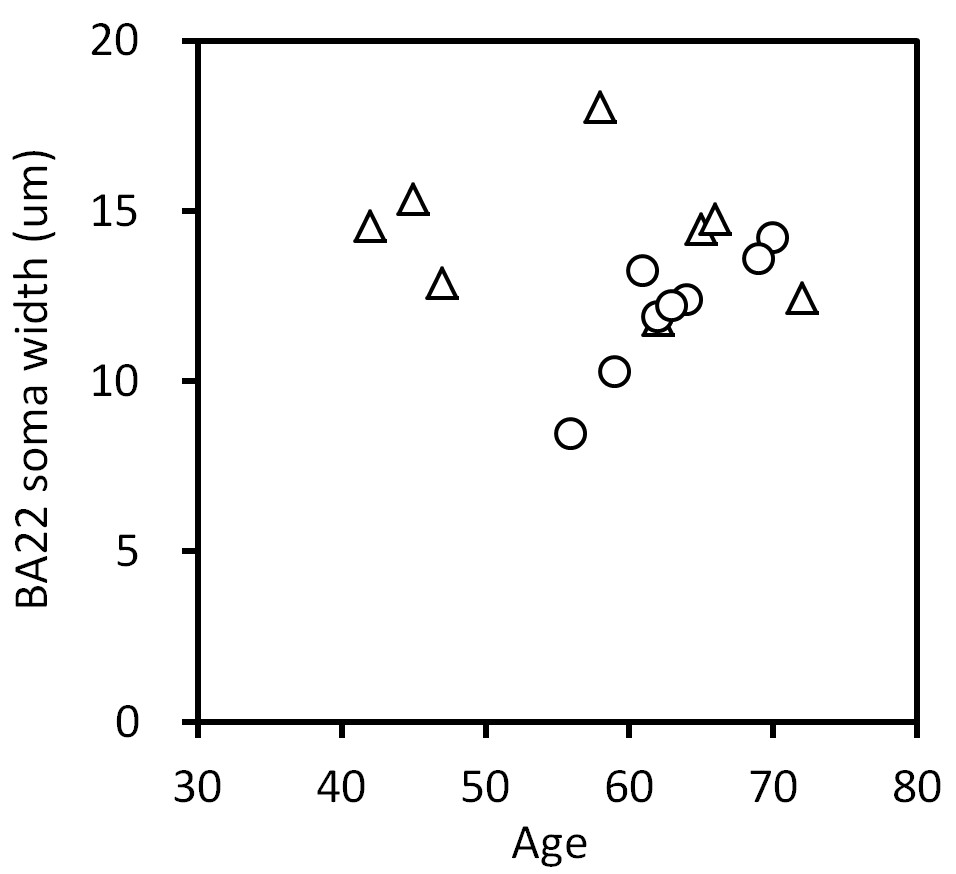

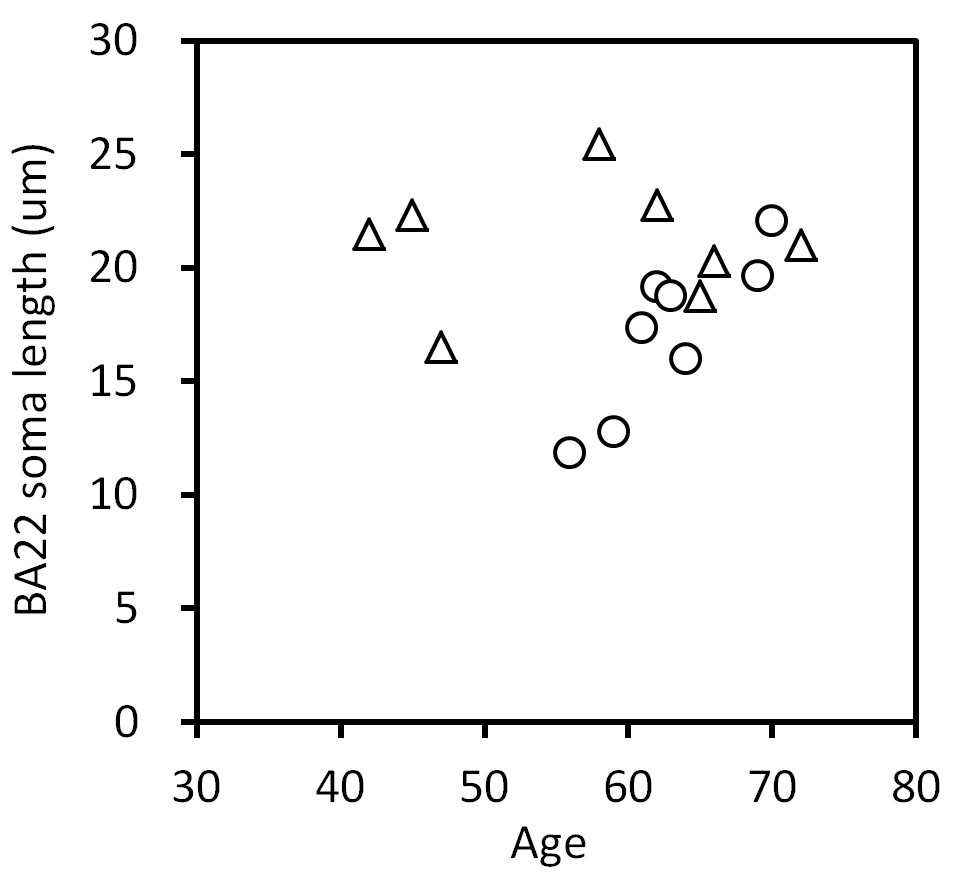


**Supplementary Figure 6.** Scatter plots of BA22 parameters versus age. Schizophrenia cases are indicated with circles and controls with triangles. (A) Neurite diameter. (B) Standard deviation of neurite curvature. (C) Spine diameter. (D) Spine length. (E) Soma width. (F) Soma length.
