# Supplementary material for: Structural alteration of neurons in schizophrenia and its relation with auditory hallucination
Source: Eur Psychiatry. 2026 Feb 6;69(1):e25. doi: 10.1192/j.eurpsy.2026.10163 (PMC12936874; doi:10.1192/j.eurpsy.2026.10163)
Supplement: Mizutani et al. supplementary material 2 — Mizutani et al. supplementary material [file S0924933826101631sup002.pdf]

Supplementary Table 1. Conditions of microtomography and nanotomography experiments.

Beamtime start date	2021.6.1	2021.11.18	2022.10.20	2011.11.17, 2012.6.18	2013.7.6 -2020.11.6
Facility	SPring-8	SPring-8	APS	SPring-8	SPring-8
Beamline	BL37XU	BL47XU	32-ID	BL20XU [1]	BL20XU [1]
X-ray energy (keV)	8.0	8.0	8.0	12.0	12.0
Contrast	Absorption	Absorption	Absorption	Absorption	Absorption
Beam condenser	Sector zone plate	Sector zone plate	CRL[2] + capillary	-	-
Outermost zone width (nm) / diameter (um) of Fresnel zone plate	100 / 310	50 / 620	50 / 180	-	-
Focal depth (um) [3]	258	65	65	-	-
Scintillator screen	P43 (Gd_2O_2S:Tb)	P43	GGG:Eu ($Gd_3Ga_5O_{12}$:Eu)	LSO (Lu_2SiO_5:Ce)	LSO, LuAG:Ce ($Lu_3Al_5O_{12}$:Ce) or GAGG:Ce ($Gd_3Al_2Ga_3O_{12}$:Ce)
Pixel size (nm)	48.1	53.7	27.5	500	500
Viewing field (pixels) [4]	2048 x 2048	2048 x 2048	2048 x 1516	1920 x 1440	2048 x 2048
Maximum image width (um)	99	110	56	960	1024
Image dynamic range (bits) [5]	14	15	14	12	12-16
Number of sample frames per dataset	1800	1800	1801	1800	1800
Degrees per frame	0.100	0.100	0.100	0.100	0.100
Exposure time per frame (msec)	200	250	500	150-200	80-200
Data collection time (sec)	800	900	1200	1080-1200	250-600
Spatial resolution (nm)	200 [6] - 250 [7]	200 [6] - 250 [7]	150 [6] - 200 [7]	1200 [6]	1200 [6]

[1] Used for visualizing overall sample structures

[2] CRL: compound refractive lens

[3] Focal depth Δf was calculated with $\Delta f = \pm \lambda / (2\ NA^2)$ and $NA = \lambda / (2\Delta r_N)$,
where λ is the wavelength, NA is the numerical aperture, and Δr_N is the outermost zone width of Fresnel zone plate.

[4] Width x height

[5] Defined from the maximum intensity of flat field images

[6] Determined using three-dimensional square-wave test patterns. These estimates represent the resolution that the instruments can reach.

[7] Determined from the Fourier domain plot. These estimates represent the resolution of the sample image itself.

Supplementary Table 2. Structural analysis summary.

Case code	S1	S2	S3	S4	S5	S6	S7	S8
Gender	female	female	male	male	female	female	female	male
Age	56	70	64	69	62	63	61	59
Schizophrenia	yes	yes	yes	yes	yes	yes	yes	yes
Onset age	26	31	21	20	50	45	18	35
Duration (year)	30	39	42	49	12	18	43	24
CP[1] equivalent dose (mg/d)	3200	1402	500	758	150	800	775	89
Hallucination score	1	1	1	2	0	2	0	1
BA22 structure								
Soma length (um) [2]	11.8 (2.3)	22.0 (5.9)	16.0 (3.7)	19.6 (7.6)	19.1 (6.9)	18.7 (10.6)	17.3 (2.6)	12.7 (2.1)
Neurite curvature (um^{-1}) [2]	1.08 (0.42)	0.56 (0.27)	0.63 (0.30)	0.36 (0.21)	0.72 (0.43)	0.65 (0.37)	0.52 (0.35)	1.03 (0.54)
Neurite radius (um) [2]	0.24 (0.20)	0.44 (0.37)	0.38 (0.22)	0.71 (0.72)	0.44 (0.50)	0.45 (0.37)	0.50 (0.41)	0.29 (0.30)
Spine curvature (um^{-1}) [2]	1.75 (0.81)	1.31 (0.60)	1.42 (0.64)	1.12 (0.55)	1.30 (0.63)	1.46 (0.74)	1.24 (0.61)	1.52 (0.74)
Spine radius (um) [2]	0.18 (0.05)	0.22 (0.06)	0.20 (0.05)	0.24 (0.06)	0.22 (0.06)	0.23 (0.07)	0.22 (0.08)	0.22 (0.06)
Spine length (um) [2]	0.90 (0.70)	1.03 (0.67)	1.12 (0.74)	0.99 (0.67)	1.27 (0.69)	0.89 (0.56)	1.05 (0.69)	0.97 (0.68)
Spine density (um^{-1}) [3]	0.166	0.181	0.190	0.169	0.223	0.170	0.166	0.105
BA24 structure[4]								
Soma length (um) [2]	20.4 (4.3)	23.2 (6.6)	24.5 (5.5)	20.4 (3.7)	19.5 (4.3)	17.8 (5.2)	18.5 (3.9)	23.2 (3.8)
Neurite curvature (um^{-1}) [2]	0.46 (0.28)	0.47 (0.32)	0.60 (0.34)	0.71 (0.36)	0.54 (0.31)	0.67 (0.46)	0.44 (0.32)	0.74 (0.42)
Neurite radius (um) [2]	0.66 (0.61)	0.63 (0.65)	0.58 (0.71)	0.39 (0.34)	0.54 (0.61)	0.43 (0.49)	0.58 (0.55)	0.42 (0.55)
Spine curvature (um^{-1}) [2]	1.13 (0.50)	1.15 (0.52)	1.19 (0.57)	1.16 (0.49)	1.40 (0.61)	1.43 (0.61)	1.32 (0.64)	1.42 (0.68)
Spine radius (um) [2]	0.23 (0.07)	0.21 (0.07)	0.22 (0.08)	0.21 (0.06)	0.21 (0.06)	0.19 (0.06)	0.22 (0.07)	0.22 (0.06)
Spine length (um) [2]	1.62 (0.82)	1.16 (0.70)	1.07 (0.69)	1.32 (0.92)	1.50 (0.79)	1.38 (0.78)	1.08 (0.58)	1.23 (0.71)
Spine density (um^{-1}) [3]	0.422	0.171	0.136	0.261	0.238	0.297	0.092	0.224

[1] Chlorpromazine

[2] Mean (standard deviation)

[3] Spine density = number of spines / total length of spiny dendrite

[4] Reproduced from Mizutani et al., 2023 and Mizutani et al., 2026

Supplementary Table 2. Structural analysis summary (cont'd).

Case code	N1	N2	N3	N4	N5	N6	N7	N8
Gender	female	female	male	male	female	male	female	male
Age	58	72	62	65	47	45	42	66
Schizophrenia	no	no	no	no	no	no	no	no
Onset age								
Duration (year)								
CP[1] equivalent dose (mg/d)								
Hallucination score								
BA22 structure								
Soma length (um) [2]	25.4 (6.6)	21.0 (2.5)	22.7 (9.6)	18.7 (7.2)	16.5 (6.2)	22.3 (6.7)	21.4 (5.5)	20.3 (4.1)
Neurite curvature (um^{-1}) [2]	0.28 (0.16)	0.58 (0.29)	0.46 (0.22)	0.35 (0.19)	0.43 (0.30)	0.34 (0.18)	0.21 (0.14)	0.34 (0.21)
Neurite radius (um) [2]	1.16 (1.22)	0.45 (0.39)	0.48 (0.37)	0.61 (0.64)	0.49 (0.43)	0.73 (0.68)	0.89 (0.67)	0.69 (0.39)
Spine curvature (um^{-1}) [2]	0.94 (0.63)	1.43 (0.61)	1.36 (0.67)	1.13 (0.59)	1.43 (0.73)	1.05 (0.63)	0.83 (0.49)	1.03 (0.53)
Spine radius (um) [2]	0.30 (0.09)	0.21 (0.06)	0.21 (0.06)	0.24 (0.07)	0.19 (0.06)	0.27 (0.08)	0.31 (0.09)	0.25 (0.08)
Spine length (um) [2]	0.85 (0.66)	1.21 (0.76)	0.81 (0.59)	0.62 (0.44)	0.86 (0.66)	0.60 (0.45)	0.73 (0.44)	1.02 (0.60)
Spine density (um^{-1}) [3]	0.047	0.284	0.168	0.079	0.122	0.084	0.050	0.256
BA24 structure[4]								
Soma length (um) [2]	26.6 (11.5)	22.8 (5.2)	28.1 (8.3)	24.3 (8.1)	26.6 (4.7)	25.0 (7.3)	30.9 (12.6)	25.0 (9.6)
Neurite curvature (um^{-1}) [2]	0.33 (0.22)	0.44 (0.21)	0.37 (0.21)	0.41 (0.23)	0.37 (0.23)	0.38 (0.25)	0.31 (0.24)	0.26 (0.19)
Neurite radius (um) [2]	1.06 (1.07)	0.53 (0.42)	0.71 (0.73)	0.59 (0.51)	0.56 (0.49)	0.64 (0.56)	0.77 (0.70)	1.02 (1.02)
Spine curvature (um^{-1}) [2]	0.80 (0.54)	1.18 (0.51)	1.16 (0.51)	1.12 (0.53)	1.40 (0.65)	1.26 (0.65)	1.23 (0.59)	1.01 (0.66)
Spine radius (um) [2]	0.29 (0.10)	0.22 (0.06)	0.20 (0.06)	0.22 (0.07)	0.19 (0.06)	0.22 (0.08)	0.23 (0.08)	0.27 (0.08)
Spine length (um) [2]	0.85 (0.80)	1.30 (0.84)	1.36 (0.77)	1.01 (0.60)	1.12 (0.65)	1.00 (0.62)	1.29 (0.74)	0.91 (0.48)
Spine density (um^{-1}) [3]	0.078	0.323	0.247	0.132	0.143	0.076	0.145	0.044

[1] Chlorpromazine

[2] Mean (standard deviation)

[3] Spine density = number of spines / total length of spiny dendrite

[4] Reproduced from Mizutani et al., 2023 and Mizutani et al., 2026

Supplementary Table 3. Statistics of datasets and Cartesian coordinate models. (**A**) Schizophrenia case S5.

Dataset name	S5A	S5B	S5C
Beamtime start date	2021.6.1	2021.6.1	2021.11.18
Image size (voxel) [1]	2060 x 2050 x 5395	2080 x 2080 x 7067	1840 x 1850 x 8177
Image size (um) [1]	99.1 x 98.6 x 259.5	100.0 x 100.0 x 339.9	98.8 x 99.3 x 439.1
Cortical depth of upper end (um)	1300	1000	2000
Number of model nodes	26341	15375	6522
Number of constituents	228	119	47
Pyramidal neurons	2	2	5
Interneurons	0	0	0
Non-typed neurons	0	1	1
Orphan neurites	225	110	40
Gliaform cells	0	3	0
Blood capillaries	1	3	1
Total length (um) [2]	8531.6	5989.9	3255.5
Pyramidal process (um)	799.8	1166.0	1048.2
Interneuron process (um)	0.0	0.0	0.0
Non-typed neuron process (um)	0.0	15.2	74.2
Orphan neurite (um)	7624.1	4375.1	2033.7
Gliaform cell process (um)	0.0	359.5	0.0
Blood capillary (um)	107.7	74.0	99.5
Number of neurite segments	300	183	102
Neurite curvature (um^{-1}) [3]	0.93 (0.36)	0.50 (0.40)	0.47 (0.32)
Neurite radius (um) [3]	0.27 (0.21)	0.61 (0.60)	0.65 (0.69)
Number of spines	631	1536	520
Spine curvature (um^{-1}) [3]	1.40 (0.66)	1.24 (0.61)	1.39 (0.62)
Spine radius (um) [3]	0.22 (0.06)	0.22 (0.06)	0.21 (0.07)
Spine length (um) [3]	1.23 (0.81)	1.29 (0.60)	1.28 (0.76)
Spine density (um^{-1}) [4]	0.123	0.359	0.198

[1] Image width x height x number of slices

[2] Spine length is not included.

[3] Mean (standard deviation)

[4] Spine density = number of spines / total length of spiny dendrite

Supplementary Table 3. Statistics of datasets and Cartesian coordinate models. (**B**) Schizophrenia case S6.

Dataset name	S6A	S6B	S6C	S6D
Beamtime start date	2021.11.18	2021.11.18	2021.11.18	2021.11.18
Image size (voxel) [1]	1930 x 1920 x 4262	1930 x 1930 x 4261	1940 x 1920 x 5572	1930 x 1920 x 5565
Image size (um) [1]	103.6 x 103.1 x 228.9	103.6 x 103.6 x 228.8	104.2 x 103.1 x 299.2	103.6 x 103.1 x 298.8
Cortical depth of upper end (um)	1700	1950	1250	900
Number of model nodes	5432	6027	10665	14552
Number of constituents	29	58	63	94
Pyramidal neurons	1	2	1	2
Interneurons	0	0	0	0
Non-typed neurons	0	1	0	0
Orphan neurites	26	52	58	91
Gliaform cells	0	1	0	0
Blood capillaries	2	2	4	1
Total length (um) [2]	2695.4	3405.9	4926.0	6160.5
Pyramidal process (um)	1616.4	905.7	1362.7	1282.0
Interneuron process (um)	0.0	0.0	0.0	0.0
Non-typed neuron process (um)	0.0	18.3	0.0	0.0
Orphan neurite (um)	944.2	1687.7	3268.2	4743.5
Gliaform cell process (um)	0.0	687.7	0.0	0.0
Blood capillary (um)	134.7	106.6	295.1	135.0
Number of neurite segments	71	89	111	174
Neurite curvature (um^{-1}) [3]	0.71 (0.35)	0.63 (0.30)	0.62 (0.44)	0.64 (0.35)
Neurite radius (um) [3]	0.41 (0.32)	0.45 (0.38)	0.53 (0.43)	0.43 (0.34)
Number of spines	233	241	566	1369
Spine curvature (um^{-1}) [3]	1.48 (0.82)	1.59 (0.83)	1.35 (0.65)	1.48 (0.75)
Spine radius (um) [3]	0.23 (0.06)	0.23 (0.06)	0.25 (0.06)	0.22 (0.07)
Spine length (um) [3]	0.66 (0.42)	0.64 (0.47)	1.03 (0.64)	0.91 (0.54)
Spine density (um^{-1}) [4]	0.103	0.108	0.143	0.239

[1] Image width x height x number of slices

[2] Spine length is not included.

[3] Mean (standard deviation)

[4] Spine density = number of spines / total length of spiny dendrite

Supplementary Table 3. Statistics of datasets and Cartesian coordinate models. (**C**) Schizophrenia case S7.

Dataset name	S7A	S7B	S7C
Beamtime start date	2021.11.18	2021.11.18	2021.11.18
Image size (voxel) [1]	1860 x 1850 x 6862	1880 x 1870 x 4266	1850 x 1860 x 5412
Image size (um) [1]	99.9 x 99.3 x 368.5	101.0 x 100.4 x 229.1	99.3 x 99.9 x 290.6
Cortical depth of upper end (um)	1700	1400	2000
Number of model nodes	11481	7568	7527
Number of constituents	129	84	75
Pyramidal neurons	1	2	2
Interneurons	0	0	1
Non-typed neurons	1	0	0
Orphan neurites	126	82	71
Gliaform cells	0	0	0
Blood capillaries	1	0	1
Total length (um) [2]	5260.1	3077.7	4098.3
Pyramidal process (um)	1094.0	490.7	1819.8
Interneuron process (um)	0.0	0.0	91.1
Non-typed neuron process (um)	28.0	0.0	0.0
Orphan neurite (um)	4124.2	2587.0	2135.4
Gliaform cell process (um)	0.0	0.0	0.0
Blood capillary (um)	14.0	0.0	51.9
Number of neurite segments	173	116	124
Neurite curvature (um^{-1}) [3]	0.50 (0.34)	0.67 (0.38)	0.40 (0.27)
Neurite radius (um) [3]	0.52 (0.49)	0.37 (0.27)	0.57 (0.39)
Number of spines	769	211	557
Spine curvature (um^{-1}) [3]	1.22 (0.60)	1.30 (0.63)	1.25 (0.61)
Spine radius (um) [3]	0.22 (0.08)	0.24 (0.08)	0.22 (0.07)
Spine length (um) [3]	1.04 (0.67)	1.10 (0.72)	1.05 (0.70)
Spine density (um^{-1}) [4]	0.193	0.111	0.164

[1] Image width x height x number of slices

[2] Spine length is not included.

[3] Mean (standard deviation)

[4] Spine density = number of spines / total length of spiny dendrite

Supplementary Table 3. Statistics of datasets and Cartesian coordinate models. (**D**) Schizophrenia case S8.

Dataset name	S8A	S8B	S8C	S8D	S8E
Beamtime start date	2021.6.1	2021.6.1	2021.6.1	2021.6.1	2021.6.1
Image size (voxel) [1]	2070 x 2100 x 7063	2070 x 2070 x 3712	2080 x 2080 x 7071	2050 x 2050 x 3711	2040 x 2040 x 5389
Image size (um) [1]	99.6 x 101.0 x 339.7	99.6 x 99.6 x 178.5	100.0 x 100.0 x 340.1	98.6 x 98.6 x 178.5	98.1 x 98.1 x 259.2
Cortical depth of upper end (um)	1150	1600	1600	1750	1800
Number of model nodes	2601	12743	17247	5777	12474
Number of constituents	5	112	85	24	127
Pyramidal neurons	1	1	2	1	1
Interneurons	0	0	0	0	0
Non-typed neurons	0	1	0	0	0
Orphan neurites	3	109	82	22	125
Gliaform cells	0	0	0	0	0
Blood capillaries	1	1	1	1	1
Total length (um) [2]	1529.5	4171.4	5701.0	2214.5	4003.7
Pyramidal process (um)	1439.5	522.4	1362.7	780.1	641.0
Interneuron process (um)	0.0	0.0	0.0	0.0	0.0
Non-typed neuron process (um)	0.0	47.0	0.0	0.0	0.0
Orphan neurite (um)	66.9	3542.3	4265.3	1402.9	3269.7
Gliaform cell process (um)	0.0	0.0	0.0	0.0	0.0
Blood capillary (um)	23.0	59.7	73.0	31.6	93.0
Number of neurite segments	37	144	123	50	144
Neurite curvature (um^{-1}) [3]	0.51 (0.33)	1.11 (0.56)	1.01 (0.50)	0.86 (0.61)	1.17 (0.47)
Neurite radius (um) [3]	0.47 (0.23)	0.27 (0.28)	0.32 (0.48)	0.33 (0.18)	0.22 (0.10)
Number of spines	185	279	460	262	186
Spine curvature (um^{-1}) [3]	1.38 (0.63)	1.53 (0.75)	1.56 (0.76)	1.57 (0.79)	1.52 (0.70)
Spine radius (um) [3]	0.24 (0.06)	0.22 (0.06)	0.21 (0.06)	0.21 (0.06)	0.22 (0.07)
Spine length (um) [3]	1.10 (0.45)	1.10 (0.99)	0.94 (0.57)	0.92 (0.64)	0.79 (0.50)
Spine density (um^{-1}) [4]	0.144	0.104	0.099	0.152	0.070

[1] Image width x height x number of slices

[2] Spine length is not included.

[3] Mean (standard deviation)

[4] Spine density = number of spines / total length of spiny dendrite

Supplementary Table 3. Statistics of datasets and Cartesian coordinate models. (**E**) Control case N5.

Dataset name	N5A	N5B	N5C	N5D	N5E
Beamtime start date	2021.6.1	2021.6.1	2021.6.1	2021.6.1	2021.6.1
Image size (voxel) [1]	2050 x 2040 x 7083	2050 x 2040 x 7077	2060 x 2050 x 8748	2050 x 2040 x 7068	2070 x 2050 x 7079
Image size (um) [1]	98.6 x 98.1 x 340.7	98.6 x 98.1 x 340.4	99.1 x 98.6 x 420.8	98.6 x 98.1 x 340.0	99.6 x 98.6 x 340.5
Cortical depth of upper end (um)	1550	1700	1800	2000	2150
Number of model nodes	4496	6928	4158	9377	7843
Number of constituents	26	47	17	46	70
Pyramidal neurons	2	3	1	3	2
Interneurons	0	1	0	0	0
Non-typed neurons	0	0	0	1	1
Orphan neurites	23	41	12	42	65
Gliaform cells	0	0	1	0	1
Blood capillaries	1	2	3	0	1
Total length (um) [2]	2540.0	4056.2	2604.3	5669.9	3979.8
Pyramidal process (um)	1709.5	2800.5	1575.6	2007.4	1379.2
Interneuron process (um)	0.0	27.5	0.0	0.0	0.0
Non-typed neuron process (um)	0.0	0.0	0.0	619.8	208.8
Orphan neurite (um)	769.8	1161.2	243.4	3042.8	2135.2
Gliaform cell process (um)	0.0	0.0	661.9	0.0	233.1
Blood capillary (um)	60.7	67.0	123.4	0.0	23.5
Number of neurite segments	85	140	45	166	131
Neurite curvature (um^{-1}) [3]	0.45 (0.34)	0.43 (0.32)	0.43 (0.31)	0.38 (0.25)	0.49 (0.30)
Neurite radius (um) [3]	0.47 (0.42)	0.48 (0.29)	0.51 (0.60)	0.52 (0.42)	0.45 (0.49)
Number of spines	104	378	240	563	465
Spine curvature (um^{-1}) [3]	1.53 (0.80)	1.46 (0.73)	1.38 (0.66)	1.40 (0.70)	1.43 (0.80)
Spine radius (um) [3]	0.20 (0.07)	0.20 (0.06)	0.18 (0.06)	0.20 (0.06)	0.19 (0.07)
Spine length (um) [3]	0.78 (0.45)	0.80 (0.77)	0.89 (0.59)	0.98 (0.72)	0.75 (0.51)
Spine density (um^{-1}) [4]	0.064	0.116	0.150	0.120	0.145

[1] Image width x height x number of slices

[2] Spine length is not included.

[3] Mean (standard deviation)

[4] Spine density = number of spines / total length of spiny dendrite

Supplementary Table 3. Statistics of datasets and Cartesian coordinate models. (**F**) Control case N6.

Dataset name	N6A	N6B	N6C	N6D	N6E
Beamtime start date	2022.10.20	2021.11.18	2021.11.18	2021.11.18	2021.11.18
Image size (voxel) [1]	1260 x 1040 x 5081	1850 x 1850 x 4258	1860 x 1840 x 4261	1870 x 1870 x 6861	1850 x 1850 x 5314
Image size (um) [1]	69.3 x 57.2 x 279.5	99.3 x 99.3 x 228.7	99.9 x 98.8 x 228.8	100.4 x 100.4 x 368.4	99.3 x 99.3 x 285.4
Cortical depth of upper end (um)	1250	1900	1700	1500	1550
Number of model nodes	1807	1649	5389	6482	6090
Number of constituents	20	28	63	57	73
Pyramidal neurons	3	1	2	2	3
Interneurons	0	0	0	0	0
Non-typed neurons	0	0	1	0	0
Orphan neurites	17	27	60	55	70
Gliaform cells	0	0	0	0	0
Blood capillaries	0	0	0	0	0
Total length (um) [2]	1479.1	1382.8	4369.2	4488.3	4924.3
Pyramidal process (um)	1108.7	586.2	1046.0	834.2	1733.9
Interneuron process (um)	0.0	0.0	0.0	0.0	0.0
Non-typed neuron process (um)	0.0	0.0	320.3	0.0	0.0
Orphan neurite (um)	370.4	796.6	3002.8	3654.2	3190.4
Gliaform cell process (um)	0.0	0.0	0.0	0.0	0.0
Blood capillary (um)	0.0	0.0	0.0	0.0	0.0
Number of neurite segments	67	61	149	114	166
Neurite curvature (um^{-1}) [3]	0.35 (0.22)	0.32 (0.17)	0.36 (0.20)	0.37 (0.18)	0.31 (0.15)
Neurite radius (um) [3]	0.71 (0.74)	0.69 (0.40)	0.77 (0.77)	0.68 (0.54)	0.75 (0.74)
Number of spines	21	75	248	383	314
Spine curvature (um^{-1}) [3]	0.90 (0.50)	0.96 (0.66)	1.00 (0.62)	1.08 (0.63)	1.09 (0.63)
Spine radius (um) [3]	0.24 (0.06)	0.29 (0.09)	0.28 (0.07)	0.26 (0.08)	0.26 (0.08)
Spine length (um) [3]	0.89 (0.51)	0.55 (0.31)	0.57 (0.38)	0.61 (0.44)	0.61 (0.52)
Spine density (um^{-1}) [4]	0.034	0.086	0.080	0.096	0.081

[1] Image width x height x number of slices

[2] Spine length is not included.

[3] Mean (standard deviation)

[4] Spine density = number of spines / total length of spiny dendrite

Supplementary Table 3. Statistics of datasets and Cartesian coordinate models. (**G**) Control case N7.

Dataset name	N7A	N7B	N7C
Beamtime start date	2021.11.18	2021.11.18	2021.11.18
Image size (voxel) [1]	1930 x 1930 x 6874	1930 x 1930 x 5570	1930x 1920 x 5571
Image size (um) [1]	103.6 x 103.6 x 369.1	103.6 x 103.6 x 299.1	103.6 x 103.1 x 299.2
Cortical depth of upper end (um)	2500	3400	2150
Number of model nodes	3519	1677	5362
Number of constituents	57	13	129
Pyramidal neurons	6	1	9
Interneurons	0	0	0
Non-typed neurons	0	0	1
Orphan neurites	51	11	119
Gliaform cells	0	0	0
Blood capillaries	0	1	0
Total length (um) [2]	4120.9	1195.4	6506.7
Pyramidal process (um)	2336.1	890.4	2023.5
Interneuron process (um)	0.0	0.0	0.0
Non-typed neuron process (um)	0.0	0.0	175.0
Orphan neurite (um)	1784.8	256.3	4308.2
Gliaform cell process (um)	0.0	0.0	0.0
Blood capillary (um)	0.0	48.8	0.0
Number of neurite segments	161	35	216
Neurite curvature (um^{-1}) [3]	0.21 (0.10)	0.41 (0.26)	0.18 (0.11)
Neurite radius (um) [3]	0.92 (0.70)	0.60 (0.31)	0.91 (0.68)
Number of spines	138	44	81
Spine curvature (um^{-1}) [3]	0.78 (0.49)	0.96 (0.50)	0.84 (0.43)
Spine radius (um) [3]	0.31 (0.09)	0.29 (0.11)	0.32 (0.09)
Spine length (um) [3]	0.78 (0.48)	0.84 (0.47)	0.59 (0.31)
Spine density (um^{-1}) [4]	0.057	0.061	0.037

[1] Image width x height x number of slices

[2] Spine length is not included.

[3] Mean (standard deviation)

[4] Spine density = number of spines / total length of spiny dendrite

Supplementary Table 3. Statistics of datasets and Cartesian coordinate models. (**H**) Control case N8.

Dataset name	N8A	N8B	N8C	N8D	N8E
Beamtime start date	2021.11.18	2021.11.18	2021.11.18	2021.11.18	2021.11.18
Image size (voxel) [1]	1860 x 1860 x 6869	1880 x 1860 x 6816	1850 x 1860 x 4264	1860 x 1860 x 4261	1850 x 1860 x 2960
Image size (um) [1]	99.9 x 99.9 x 368.9	101.0 x 99.9 x 366.0	99.3 x 99.9 x 229.0	99.9 x 99.9 x 228.8	99.3 x 99.9 x 159.0
Cortical depth of upper end (um)	1950	1500	1950	1750	1400
Number of model nodes	11461	7766	3011	4729	3694
Number of constituents	63	73	15	28	32
Pyramidal neurons	3	2	1	1	3
Interneurons	1	0	0	0	0
Non-typed neurons	0	0	0	0	1
Orphan neurites	58	69	14	27	27
Gliaform cells	0	1	0	0	1
Blood capillaries	1	1	0	0	0
Total length (um) [2]	6484.2	4266.6	1849.5	2135.9	2267.4
Pyramidal process (um)	3123.6	1457.7	1236.6	1018.1	792.8
Interneuron process (um)	749.9	0.0	0.0	0.0	0.0
Non-typed neuron process (um)	0.0	0.0	0.0	0.0	372.2
Orphan neurite (um)	2582.8	2294.4	613.0	1117.8	1005.2
Gliaform cell process (um)	0.0	470.8	0.0	0.0	97.2
Blood capillary (um)	27.9	43.8	0.0	0.0	0.0
Number of neurite segments	168	149	53	61	84
Neurite curvature (um^{-1}) [3]	0.35 (0.21)	0.34 (0.23)	0.30 (0.12)	0.38 (0.22)	0.29 (0.18)
Neurite radius (um) [3]	0.64 (0.36)	0.73 (0.43)	0.64 (0.25)	0.61 (0.24)	0.83 (0.51)
Number of spines	1060	1022	354	641	451
Spine curvature (um^{-1}) [3]	1.06 (0.51)	1.02 (0.55)	1.01 (0.55)	1.01 (0.51)	1.07 (0.54)
Spine radius (um) [3]	0.24 (0.07)	0.26 (0.08)	0.25 (0.08)	0.26 (0.07)	0.23 (0.08)
Spine length (um) [3]	1.02 (0.63)	1.03 (0.58)	0.98 (0.61)	1.03 (0.57)	1.03 (0.61)
Spine density (um^{-1}) [4]	0.202	0.335	0.216	0.315	0.246

[1] Image width x height x number of slices

[2] Spine length is not included.

[3] Mean (standard deviation)

[4] Spine density = number of spines / total length of spiny dendrite
